# Expansion microscopy applied to mono- and dual-species biofilms

**DOI:** 10.1038/s41522-023-00460-x

**Published:** 2023-12-05

**Authors:** David Valdivieso González, Josué Jara, Víctor G. Almendro-Vedia, Belén Orgaz, Iván López-Montero

**Affiliations:** 1 Dto. Química Física, Universidad Complutense de Madrid, Avda. Complutense s/n, 28040 Madrid, Spain; 2 Instituto Pluridisciplinar, Universidad Complutense de Madrid, Ps. Juan XXIII 1, 28040 Madrid, Spain; 3 Instituto de Investigación Biomédica Hospital Doce de Octubre (Imas12), Avda. de Córdoba s/n, 28041 Madrid, Spain; 4 Sección Departamental de Nutrición y Ciencia de los Alimentos, Facultad de Veterinaria, Universidad Complutense de Madrid, 28040 Madrid, Spain; 5 Sección Departamental de Farmacia Galénica y Tecnología Alimentaria, Facultad de Veterinaria, Universidad Complutense de Madrid, Avda. Complutense s/n, 28040 Madrid, Spain; 6 Present Address: Instituto de Investigación Biomédica Hospital Doce de Octubre (Imas12), Avda. de Córdoba s/n, 28041 Madrid, Spain

**Keywords:** Biological techniques, Biofilms

## Abstract

Expansion microscopy (ExM) is a new super-resolution technique based on embedding the biological sample within a hydrogel and its physical expansion after swelling. This allows increasing its size by several times while preserving its structural details. Applied to prokaryotic cells, ExM requires digestion steps for efficient expansion as bacteria are surrounded by a rigid cell wall. Furthermore, bacteria can live in social groups forming biofilms, where cells are protected from environmental stresses by a self-produced matrix. The extracellular matrix represents an additional impenetrable barrier for ExM. Here we optimize the current protocols of ExM and apply them to mono- and dual-species biofilms formed by clinical isolates of *Limosilactobacillus reuteri*, *Enterococcus faecalis*, *Serratia marcescens* and *Staphylococcus aureus*. Using scanning electron microscopy for comparison, our results demonstrate that embedded bacteria expanded 3-fold. Moreover, ExM allowed visualizing the three-dimensional architecture of the biofilm and identifying the distribution of different microbial species and their interactions. We also detected the presence of the extracellular matrix after expansion with a specific stain of the polysaccharide component. The potential applications of ExM in biofilms will improve our understanding of these complex communities and have far-reaching implications for industrial and clinical research.

## Introduction

Super-resolution microscopy techniques have revolutionized in the last decades our understanding of biological systems. A deep knowledge of light properties and electronics allowed the development of X-ray crystallography, electron microscopy, atomic force microscopy or advanced fluorescence microscopies such as STORM or STED, among others^1–3^. All of them achieve imaging above optical resolution of visible light eventually reaching the single-molecule level. However, these techniques often require specialized equipment and expertise, which can limit their accessibility and scalability to complex systems. Recently, sample preparation was considered as a new strategy for enhancing resolution using conventional fluorescence microscopy by physically expanding biological specimens, such as a cell or tissue, in polymeric hydrogels, leading to the termed Expansion Microscopy (ExM)^4^.

In ExM, biomolecules, such as proteins within a cell, are covalently anchored to a polymer network that expands isotropically after swelling. As a result, the biological sample increases its size while keeping the relative distance among the constitutive components, as imaged through fluorescent probes^4^. ExM has undergone significant improvements since it was first introduced in 2015. On one hand, new methods have extended the use of ExM for the specific staining of proteins and organelles using genetically encoded fluorescent tags or fluorescent antibodies^5–7^. Also, RNA imaging at the single-molecule level has been shown^8^. On the other hand, consecutive gel expansions and enhanced resolutions have been achieved^9^. Additionally, the combination of ExM with click chemistry (click-ExM) contributed to enlarge the number of biomolecules suitable for ExM, such as lipids, glycans, proteins, DNA, RNA and small molecules^10^ that were not preserved after expansion applying original protocols.

Although ExM has been mainly applied to study the subcellular organization of eukaryotic cells, ExM has also progressed in prokaryotic expansion^11–13^. Bacterial samples are intrinsically unsusceptible to ExM as they are surrounded by the fairly rigid cell wall, which needs an additional digestion step for a homogeneous and efficient expansion^14,15^. One of the most common enzymes used to disrupt bacterial cell walls is lysozyme, which breaks down the polysaccharide chains in the peptidoglycan layer of the cell wall. Lysozyme is commonly used in combination with detergents and other agents to facilitate the penetration of the enzyme into the cell wall. Due to differences in cell wall among microorganisms, bacterial ExM yields non-uniform expansions^14,15^.

Here we apply ExM to bacterial biofilms. Biofilms are surface-associated complex communities of microorganisms embedded on a self-produced structured matrix of extracellular polymeric substances (EPS)^16,17^. The EPS matrix represents 90% of the total biofilm biomass and is mainly formed by water and soluble factors as polysaccharide biopolymers, proteins or extracellular DNA (eDNA) among others, that facilitate the adhesion of the cells to each other or/and to the surface^18–20^. The EPS matrix provides mechanical support and stability to the biofilm, allowing it to resist shear forces and other mechanical stresses^21^. The matrix also creates a barrier that prevents the diffusion of nutrients and waste products, precluding antibiotics and other antimicrobial agents from penetrating the biofilm^22,23^. As a result, the EPS matrix might represent an additional impenetrable barrier for ExM.

**Fig. 1:**
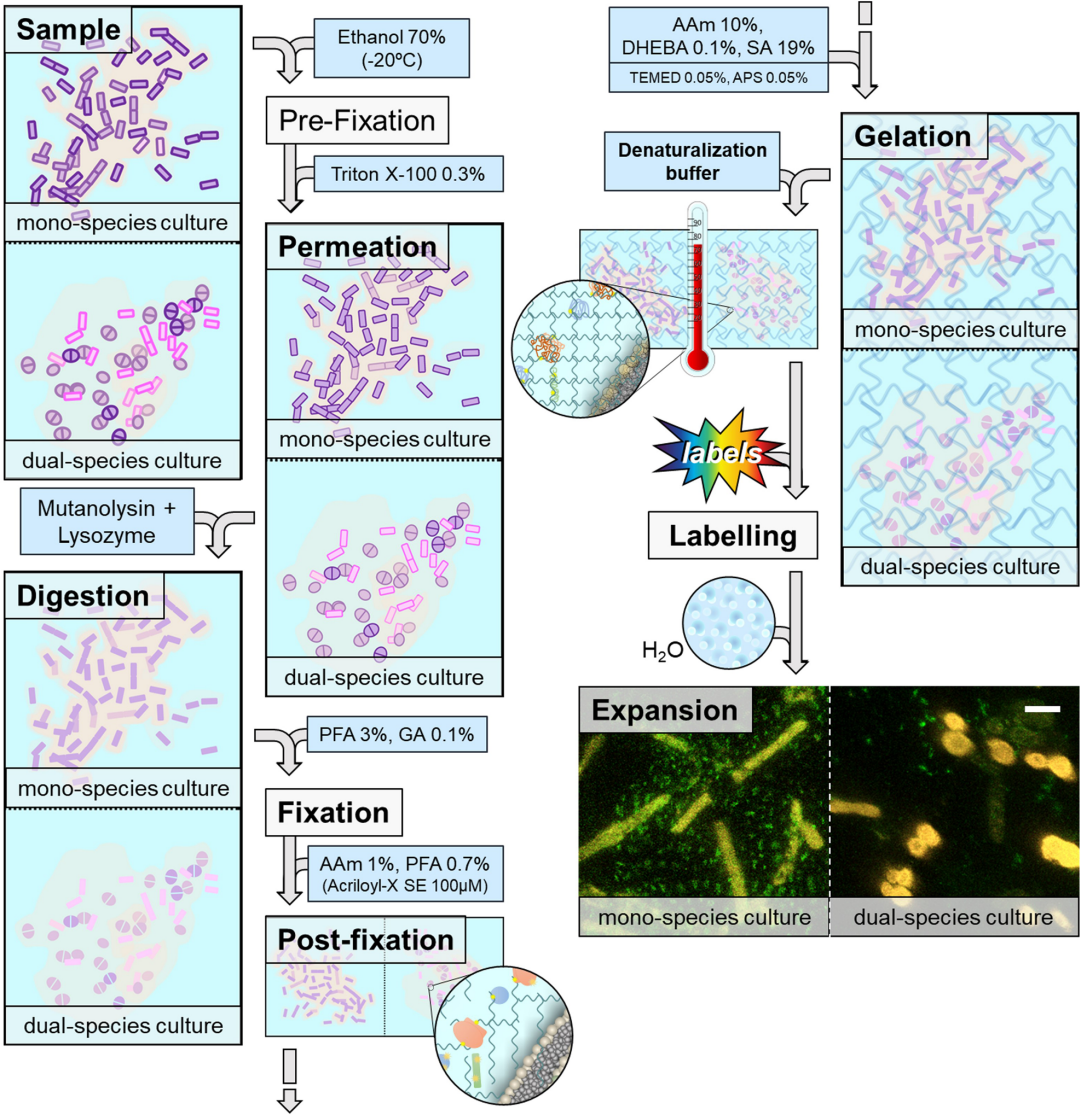
Biofilm expansion microscopy workflow. Biofilm samples are pre-fixed with ethanol 70% followed by a soft detergent permeation and an enzymatic digestion. Mutanolisin + lysozyme digestion ensures isotopic expansion of the different bacterial species, where the matrix also expands. After digestion, expansion follows similarly to pan-ExM^9^ up to homogenization where both a denaturation buffer and high temperature denaturalize proteins and allow the subsequent gel expansion. Finally, denaturalized gels are labelled with fluorescent probes and expanded in water. Expansion micrographs correspond to *Limosilactobacillus reuteri* as representative for mono-species biofilms and to *Serratia marcescens* + *Staphylococcus aureus* as representative for a dual-species biofilm. Proteome is labelled in red whereas the polysaccharide component of the EPS matrix is labelled in green. Scale bar: 5 µm.

Despite the complex structure of biofilms, we improved the current protocols of ExM with an additional digestion step based on the combined action of lysozyme with mutanolysin, which breaks down the glycan strands of peptidoglycan^14^ while they preserve the biofilm architecture. This enzyme is particularly effective against gram-positive bacteria, which have a thicker peptidoglycan layer than gram-negative bacteria. Our results show that ExM can be applied to mono- and dual-species biofilms produced by clinical isolates of *Limosilactobacillus reuteri*, *Enterococcus faecalis*, *Serratia marcescens* and *Staphylococcus aureus* (Fig. 1). By comparison with scanning electron microscopy, we show that bacteria experienced a ≈3-fold expansion, preserving the three-dimensional architecture of the biofilm and the distribution of different microbial species and their interactions within the EPS matrix. Additionally, we also report evidence about the detection of the polysaccharide component of the matrix within the biofilm.

The ability to study the structure and properties of biofilms with high resolution and accuracy using ExM could greatly improve our understanding of these complex systems and contribute for developing new strategies to prevent and treat biofilm-associated infections, which are a significant clinical and environmental problem.

## Results

### Biofilm expansion requires the combined digestion of lysozyme and mutanolysin

**Fig. 2:**
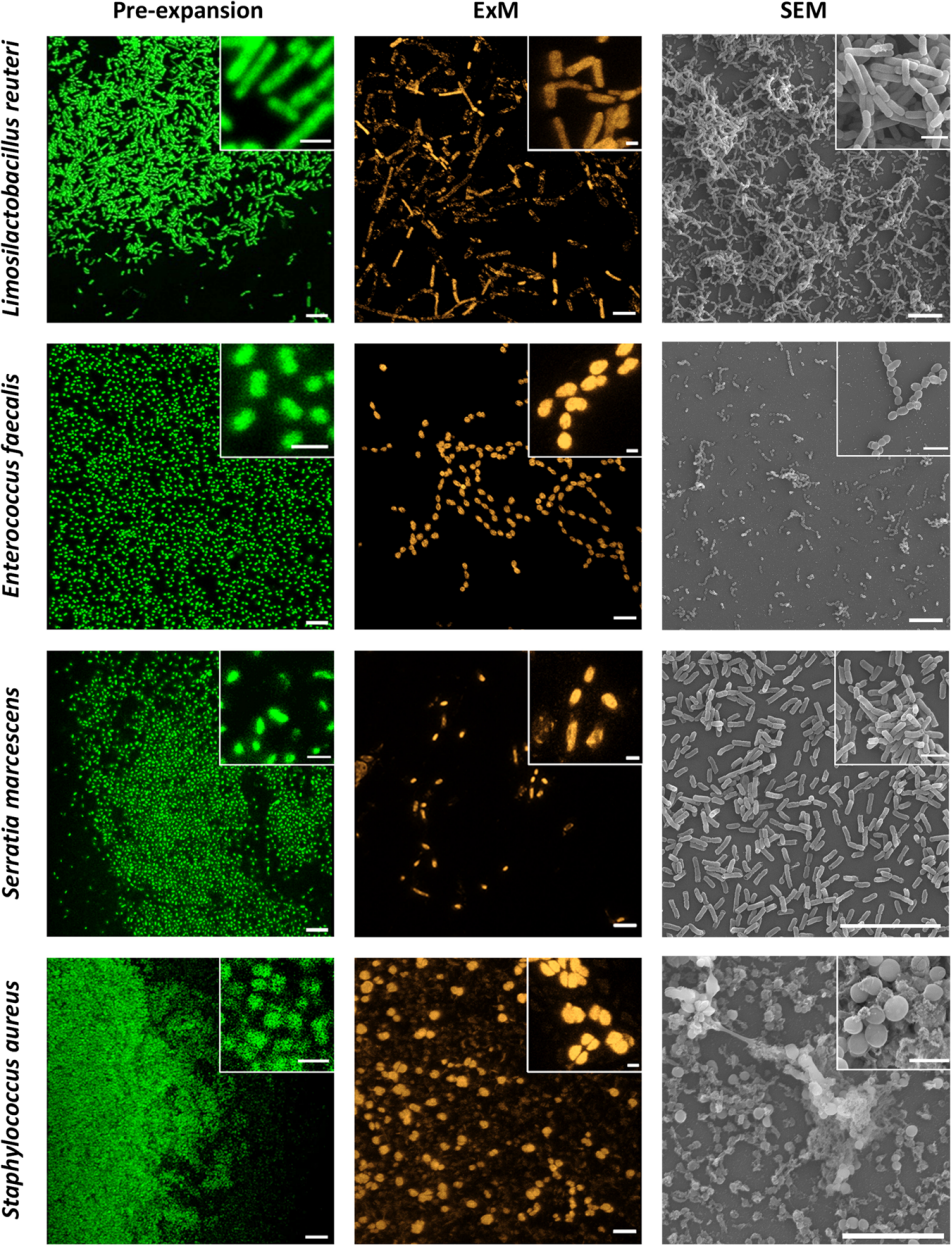
Representative images of mono-species biofilms (48 h) of *Limosilactobacillus reuteri*, *Enterococcus faecalis*, *Serratia marcescens* and *Staphylococcus aureus*. **Left** and **middle**. Pre-expanded and expanded biofilms as visualized by CLSM. Pre-expanded and expanded biofilms were labelled with SYTO 13 and Alexa Fluor 555 NHS ester, respectively. Scale bars: 10 μm. Inset scale bars: 2 μm. Expansion factors are 2.5, 3.6, 2.6 and 2.8 for *L. reuteri*, *E. faecalis*, *S. marcescens* and *S. aureus*, respectively. **Right**. Non-expanded biofilms as visualized by SEM. Magnifications are ×1000 (×9000, inset) for *L. reuteri* and *E. faecalis;* ×3000 (×5000, inset) for *S. marcescens* and ×3000 (×9000, inset) for *S. aureus*.

Prokaryotic expansion protocols need a digestion step before fixation and gelation of the sample (see Methods and Fig. 1). The enzymatic digestion allows the breakdown of the bacterial cell wall for further internalization of the hydrogel network and fluorescent probes^12,14,15^. To check whether prokaryotic expansion protocols were straightforward applied for studying biofilms, we developed 12 h and 48 h mono-species biofilms of *L. reuteri*, *E. faecalis*, *S. marcescens* and *S. aureus*. These species are representative for different gram-stain and bacterial shapes. Remarkably, lysozyme digestion (5 kU/mL) resulted into non-homogeneous expansions. Whereas some of the bacteria expanded completely, crescent-like shapes were observed in *L. reuteri* biofilms (Supplementary Fig. 1). The heterogeneous expansion might be due to a partial digestion of the cell wall^14^. To improve the digestion step we used mutanolysin as an alternative enzyme for processing the cell wall peptidoglycan. Unlike young biofilms (12 h) (Supplementary Fig. 2*)*, mature biofilms (48 h) were not embedded into the acrylamide gel and thus non-adapted to ExM, suggesting a low capability of mutanolysin to penetrate the EPS matrix. To fully digest the bacterial cell wall, an improved digestion step was required. A mixture of both enzymes, lysozyme (5 kU/mL) and mutanolysin (160 U/mL), was added simultaneously and incubated overnight. Note that a 4-fold increase in the enzymes concentration did not result into better expansions (Supplementary Fig. 2). The combined action of lysozyme and mutanolysin resulted into homogeneous expansions (Fig. 2) in the absence of any other specific EPS-digestion enzyme.

### Biofilm expansion preserves bacteria morphology and their three-dimensional organization

To evaluate the impact of the expansion treatment on the integrity of biofilms we visually examined both the bacteria morphology and the three-dimensional organization of cells within the biofilm before and after expansion using confocal laser scanning fluorescence microscopy (CLSM) and compared them with non-expanded biofilms visualized with scanning electron microscopy (SEM) (Fig. 2). *L. reuteri* is a rod-shaped gram-positive bacterium (~0.65 μm width and ~1.5 μm long) that forms dense biofilms in which cells are organized forming a complex network. ExM images revealed in detail the network structure formed by expanded cells (Fig. 2). *E. faecalis* are cocci (~0.60 μm diameter) gram-positive bacteria, forming monolayer biofilms. Both ExM and SEM images confirm that *E. faecalis* cells maintain the original disposition into linear arrangements with a bigger interspace due to expansion (Fig. 2). *S. marcescens* are motile, short rod-shaped (~0.50 μm width and ~1.2 μm long) gram-negative bacteria which form small and dispersed clusters (Fig. 2). Again, ExM images reveal that cells maintained their original shape and their capability to form small groups of 4–5 cells. *S. aureus* are immobile spherical (~0.80 μm diameter) gram-positive bacteria. Their biofilms are organized in dense clusters of cells, generally pairwise organized, surrounded and interconnected by the EPS matrix (Fig. 2). Surprisingly, ExM images also revealed the arrangement of cells into diplococcus with an enhanced contrast between cells due to expansion.

### Biofilm expansion is isotropic

**Fig. 3:**
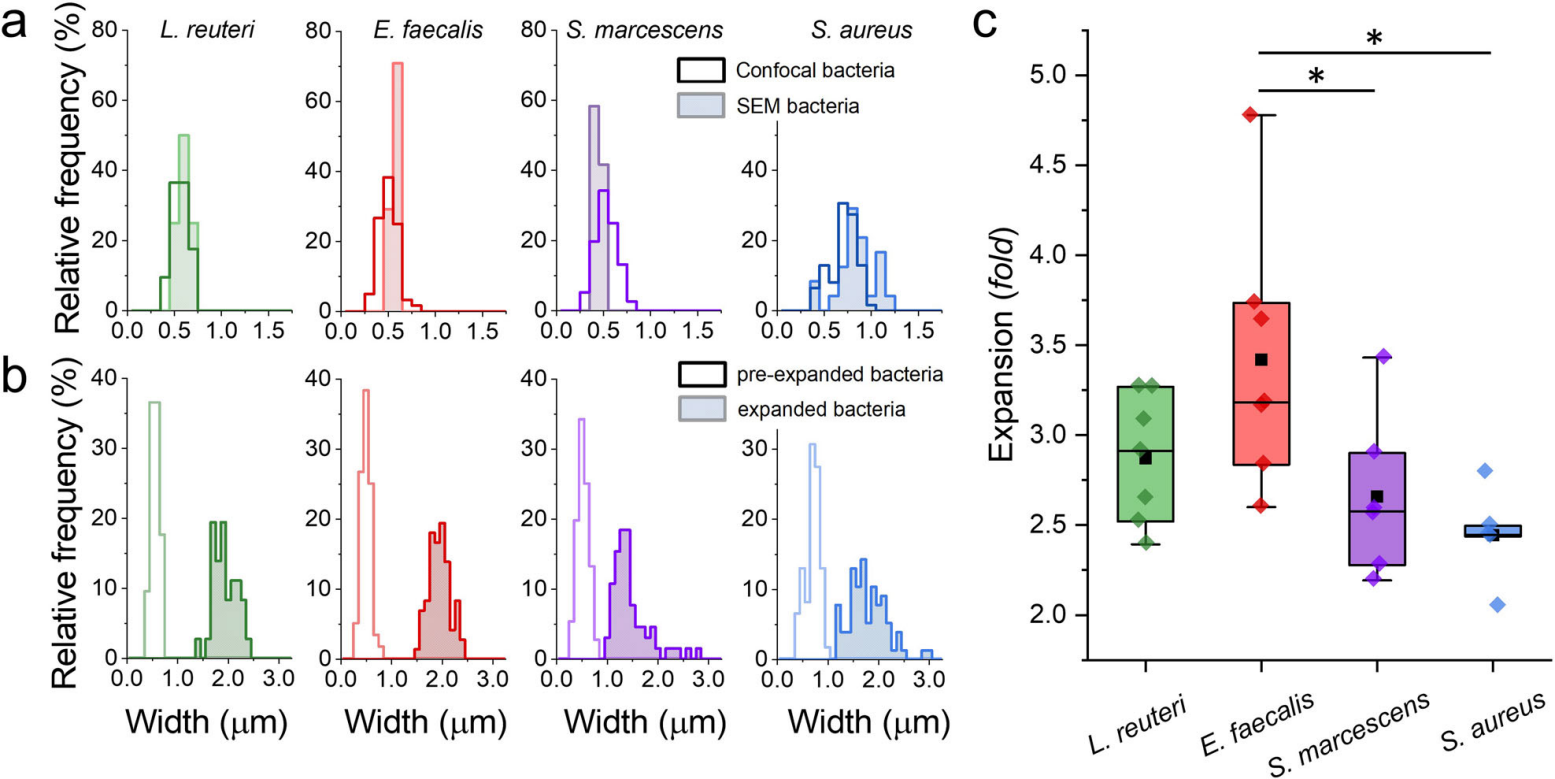
Expansion factors of mono-species biofilms. **a** Relative frequency of bacterial width of non-expanded biofilms as measured from confocal images (empty field) and SEM images (coloured field). **b** Relative frequency of bacterial width of non-expanded (empty field) and expanded (coloured field) biofilms as measured from confocal images. **c** Expansion factors of mono-species biofilms. Average values ± standard deviations are 3.0 ± 0.3 (*n* = 7) for *L. reuteri*, 3.4 ± 0.7 (*n* = 7) for *E. faecalis*, 2.6 ± 0.4 (*n* = 7) for *S. marcescens* and 2.4 ± 0.3 (*n* = 5) for *S. aureus*. Statistical significance: *(*p*-value < 0.05; Tukey Mean Difference test). Boxplot elements are black square-mean; centre line-median; box limits-upper and lower quartiles; whiskers-extreme values; colour diamonds: experimental data.

A further validation of the biofilm expansion protocol was assessed with a quantitative measurement of the expansion factor. To calculate an isotropic expansion of the sample, we independently evaluated and compared a linear expansion (1D) factor ($F_{1D}^{ExM}$) and a volumetric expansion (3D) factor ($F_{3D}^{ExM}$) of individual bacterial cells within the biofilm. Linear expansion factors, $F_{1D}^{ExM}$, were measured as the increment of the bacterial width between expanded and pre-expanded samples using CLSM and SEM (Fig. 3a, b), whereas volumetric expansion factors, $F_{3D}^{ExM}$, were obtained as the cube-root of the three-dimensional reconstructed volume ratio from individual cells before and after the expansion treatment (Supplementary Fig. 3 and Supplementary Table 1). Note that the expanded volume scales with the pre-expanded volume through a cubic factor λ^3^ ^24^. Notably, we find a good correlation between $F_{1D}^{ExM}$ and $F_{3D}^{ExM}$ values (Supplementary Table 1). Whereas $F_{1D}^{ExM}$ values were 3.0 ± 0.3, 3.4 ± 0.7, 2.6 ± 0.4 and 2.4 ± 0.3; $F_{3D}^{ExM}$ values were 2.95 ± 0.19, 2.52 ± 0.03, 3.06 ± 0.08 and 2.30 ± 0.1 for *L. reuteri* (*n* = 7), *E. faecalis* (*n* = 7), *S. marcescens* (*n* = 7) and *S. aureus* (*n* = 5), respectively. The similar value for both expansion factors is indicative for a homogeneous and isotropic expansion.

### Biofilm expansion factor depends on bacterial species

Our results also indicate that expansions factors depend on bacterial species (Fig. 3c and Supplementary Table 2). Differences in the expansion efficiency might be related with the shape and gram-stain of bacteria. However, rod-shaped bacteria, i.e.*, L. reuteri* and *S. marcescens* exhibited statistically similar expansion factors, despite being gram-positive and gram-negative bacteria, respectively. Unlike rod-shaped bacteria, we obtained statistically significant differences in expansion factors (*p* < 0.05) from spherical-shaped and gram-positive bacteria. Certainly, *S. aureus* cells expanded less than *E. faecalis* cells. As revealed by SEM images (Fig. 2), *S. aureus* produces biofilms with a higher content of EPS matrix. As the enzymatic digestion might be less efficient in this case, we increased the mutanolysin and lysozyme concentrations (up to 4-fold) to detect an impact on the expansion factor. However, higher concentrations of mutanolysin and lysozyme did not increase the expansion of *S. aureus* biofilms, which are characterized by an expansion factor close to 2.4 (Supplementary Fig. 3). Overall, biofilm expansion is generally characterized by an expansion factor close to 3.0-fold, independently of the concentration on the digestion enzymes.

### Expansion preserves the relative position of single cells within the three-dimensional architecture of biofilm

**Fig. 4:**
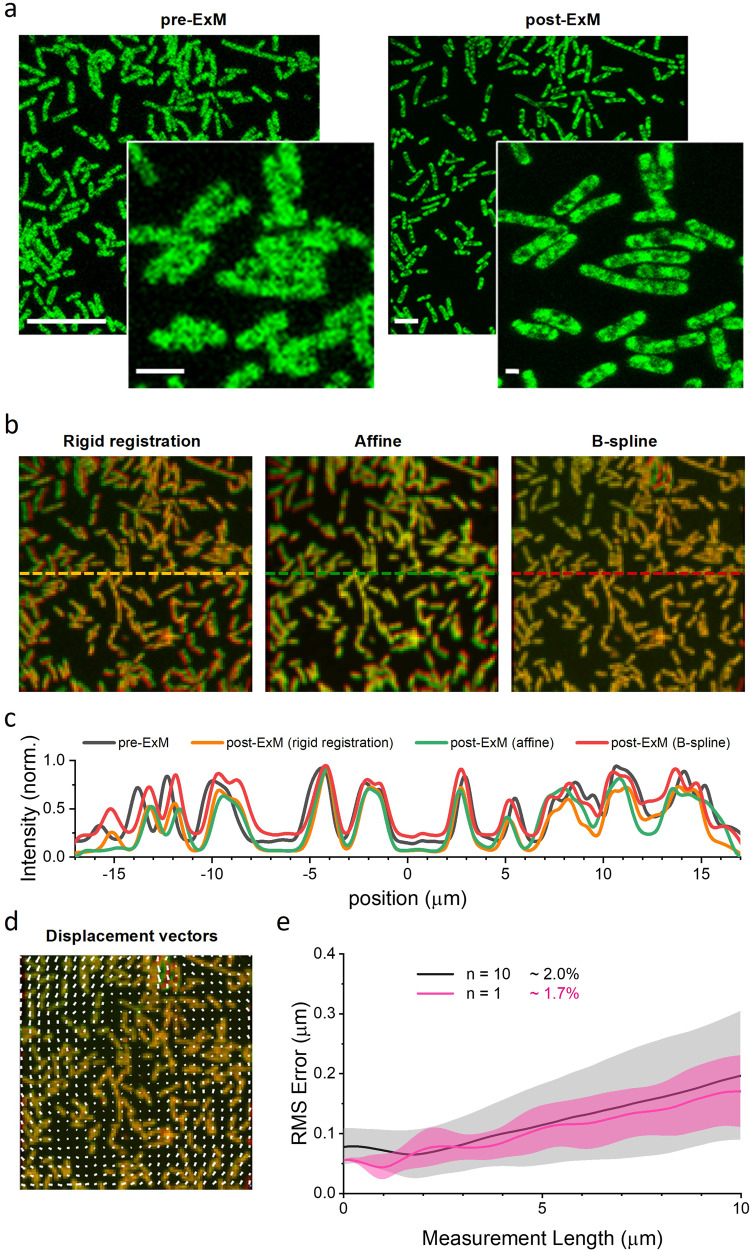
Preservation of the relative position of single cells within the three-dimensional architecture of biofilm after expansion. **a** Representative images of low and high magnification fields of pre-expanded (**left**) and post-expanded (**right)** sample of a *L. reuteri* biofilm. Both samples were labelled with Alexa Fluor 488 NHS ester. Scale bars: 10 μm. Zoom scale bars: 2 μm. Expansion factor is 3.0. **b** Composite fluorescence image of pre-expanded and rigid (**left**), affine (**middle**) and B-spline (**right**) registered post-expanded images, respectively. **c** Line intensity profile along the dashed lines shown in (**b**). **d** Distortion vector field (white arrows) of pre-expanded and B-spline registered post-expanded images. **e** Root mean square (RMS) error over length measurement comparing pre- and post-expanded images obtained from the deformation vector field after the B-spline registration.

A conclusive proof for the preservation of the three-dimensional structure of biofilms upon expansion was obtained by the distortion quantification of relative distances among single bacteria within the same field of view before and after expansion. For this, we developed a biofilm using *L. reuteri* as model microorganism (Fig. 4a). A first alignment of pre- and post-expanded images from the same region of interest was achieved using rigid image registration. Next, a sequential processing of post-expanded images was performed using affine and B-spline-based non-rigid registrations (Fig. 4b). Changes in cell position before and after expansion were obtained from the line intensity profiles, which presented an improved overlap after distortion correction (Fig. 4c). Finally, we obtained the deformation vector field from the B-spline registration (Fig. 4d) and quantified the root-mean-square (RMS) error of the relative distances among different bacteria in a 10-µm length-scale. As previously described by other authors using different ExM protocols^9,25^, we found that RMS errors at that length scale were ~2% indicating that our expansion protocol preserves the relative position of cells within the biofilm architecture (Fig. 4e).

### Biofilm expansion presents a limited preservation of nanoscale information

**Fig. 5:**
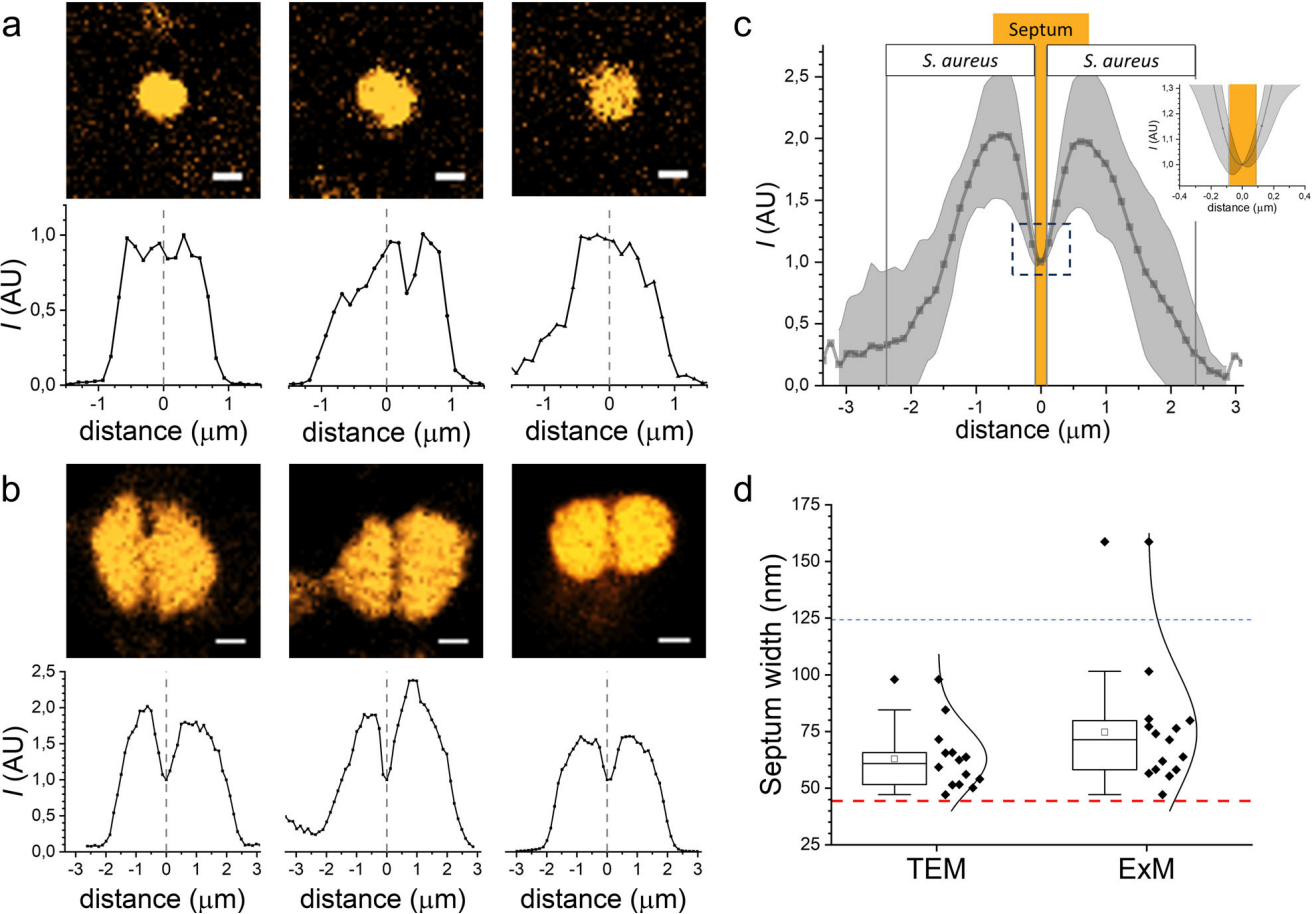
Nanoscale preservation of ExM as validated by the measurement of the division septum in *S. aureus*. **a Top:** Pre-expansion images of pairwise bacteria in *S. aureus* biofilms. Proteome was labelled with Alexa Fluor 555 NHS ester. Scale bars: 1 μm. **Bottom:** Averaged intensity profiles along *x*-direction of cells shown above. Zero distance is set at the centre of bacteria. **b Top:** ExM images of pairwise bacteria in *S. aureus* biofilms. Proteome was labelled with Alexa Fluor 555 NHS ester. Scale bars: 1 μm (${{{\mathrm{F}}}}_{1{{{\mathrm{D}}}}}^{{{{\mathrm{ExM}}}}} \approx 2.8$). **Bottom:** Averaged intensity profiles in the orthogonal direction of septa of pairwise cells shown above. Zero distance is set at the septum of bacteria. **c** Mean intensity profile ± standard deviation in the orthogonal direction of septa from different pairwise cells (*n* = 15). The septum is expected to be localized within three consecutive pixels centred at the minimum intensity value (orange box, see main text for details). **d** Box plots of the septum width as measured by TEM^26–28^ (**left**) and ExM (**right**). The blue line represents the pixel size of the microscope (~125 nm/pixel with a Plan Apo 100× NA 1.45 oil immersion objective, Nikon). The red line represents the pixel size after expansion (~125/2.8 = 45 nm/pixel). Note that outlier values might be due to septa from bacteria in a late state of cell division where the septum might be wider). Boxplot elements are: white square-mean; centre line-median; box limits-upper and lower quartiles; whiskers-extreme values; diamonds: experimental data; curved line: normal distribution.

A first validation of ExM at nanoscale was addressed by measuring the division septum on expanded *S. aureus* biofilms (using sample 4 in Supplementary Table 4, with a $F_{1D}^{ExM} = 2.8$). Unlike conventional CLSM of untreated biofims, ExM is able to resolve the septum in *S. aureus* (Fig. 5a, b). During cell division, the septum spatial localization and progression are mainly regulated by the cell wall architecture. As peptidoglycan is the major component of the cell wall, the proteome labelling used in ExM displays the nascent cells as individuals units separated by a non-fluorescent banded surface (Fig. 5b). Averaged intensity profiles in the orthogonal direction of different septa clearly show the absence of protein labelling across the division plane (Fig. 5b, bottom). The width of the complete division septum in *S. aureus* is a highly conserved nanostructure, with typical dimensions of 63 ± 14 nm (*n* = 14), as measured from electron micrographs reported in different publications^26–28^. The width is thus well below the Abbe resolution limit (~190 nm at λ = 555 nm) and pixel size (~125 nm/pixel) of our microscope using a Plan Apo 100× NA 1.45 oil immersion objective (Nikon). After expansion, the resolution limit and the pixel sized increased up to ~200/2.8 = 70 nm and ~45 nm/pixel, respectively. Under these conditions, the width of the septum should extend over three consecutive pixels centred at the minimum intensity value, as shown by the intensity profile of 15 averaged septa from independent cells (Fig. 5c). To obtain the actual width of individual septa we measured the distance of the non-fluorescent band, yielding a value of 75 ± 27 nm (*n* = 15) (Fig. 5d). The obtained averaged value supports the nanoscale validation of ExM.

An additional assessment of nanoscale preservation upon expansion was evaluated by imaging the well-known ring structure of FtsZ division protein (Supplementary Fig. 4). Here, we used the strain *Escherichia coli* 8H3-10 for the specific immunolabelling of FtsZ with an anti-FtsZ polyclonal antiserum and a secondary Alexa555-conjugated anti-rabbit antibody. Before expansion (Supplementary Fig. 4a), biofilms were characterized by small and dispersed clusters containing rod-shaped bacteria (~0.56 nm width and ~1.2 nm long, *n* = 30) as revealed by the blurred fluorescence intensity from unspecific proteome labelling (Alexa Fluor 488 NHS, green channel). As expected, the fluorescence immunolabelling revealed the presence of FtsZ proteins found at 2 main locations, i.e. the poles and the midpoints of *E. coli* cells as shown by the intensity profiles along the cells (Supplementary Fig. 4b). As cells in biofilms do not exhibit balanced microbial growth, the metabolic state of individual bacteria is very heterogeneous displaying different location of FtsZ. After expansion (Supplementary Fig. 4c), cells maintained their original shape and their capability to form small clusters. A linear expansion factor $F_{1D}^{ExM} \approx 4$ was obtained. Interestingly, the unspecific proteome labelling (green channel) showed a very intense and well defined structure (Supplementary Fig. 4d, e), which partially reached the poles of cells, suggesting a plausible labelling of nucleoid, which is enriched in polymerases and transcription machineries. As for FtsZ proteins, the specific immunolabelling showed the expected location of proteins within the cell, including the division site and the poles (Supplementary Fig. 4d, e), representative of different steps of the FtsZ cycle during cell division (Supplementary Fig. 4f)^29^. Unfortunately, the immunolabelling quality presented here was not suitable enough compared with previously reported FtsZ staining data using an alternative ExM protocol^13^. Here, the stained proteome became long and narrow after expansion and the immunostaining pattern for FtsZ presented a non-specific accumulation of anti-FtsZ antibody at the periphery of cells. The proof of concept nature of these particular experiments shows the limited ability of the adapted ExM protocol for specific labelling of protein structures within the cell.

### Bacterial communities are conserved in dual-species biofilms after expansion

Real biofilms can be composed of tens of different bacterial strains that coexist within the same EPS matrix. The potential application of ExM in biofilms issued from a wide range of natural and industrial environments, such as soil, water or medical devices, requires the ability of maintaining after expansion the structural and compositional features of complex bacterial communities. We then grew 6 dual-species biofilm systems (BS_i_) as a simple model by combining the 4 strains used above (*see* Supplementary Table 3).

**Fig. 6:**
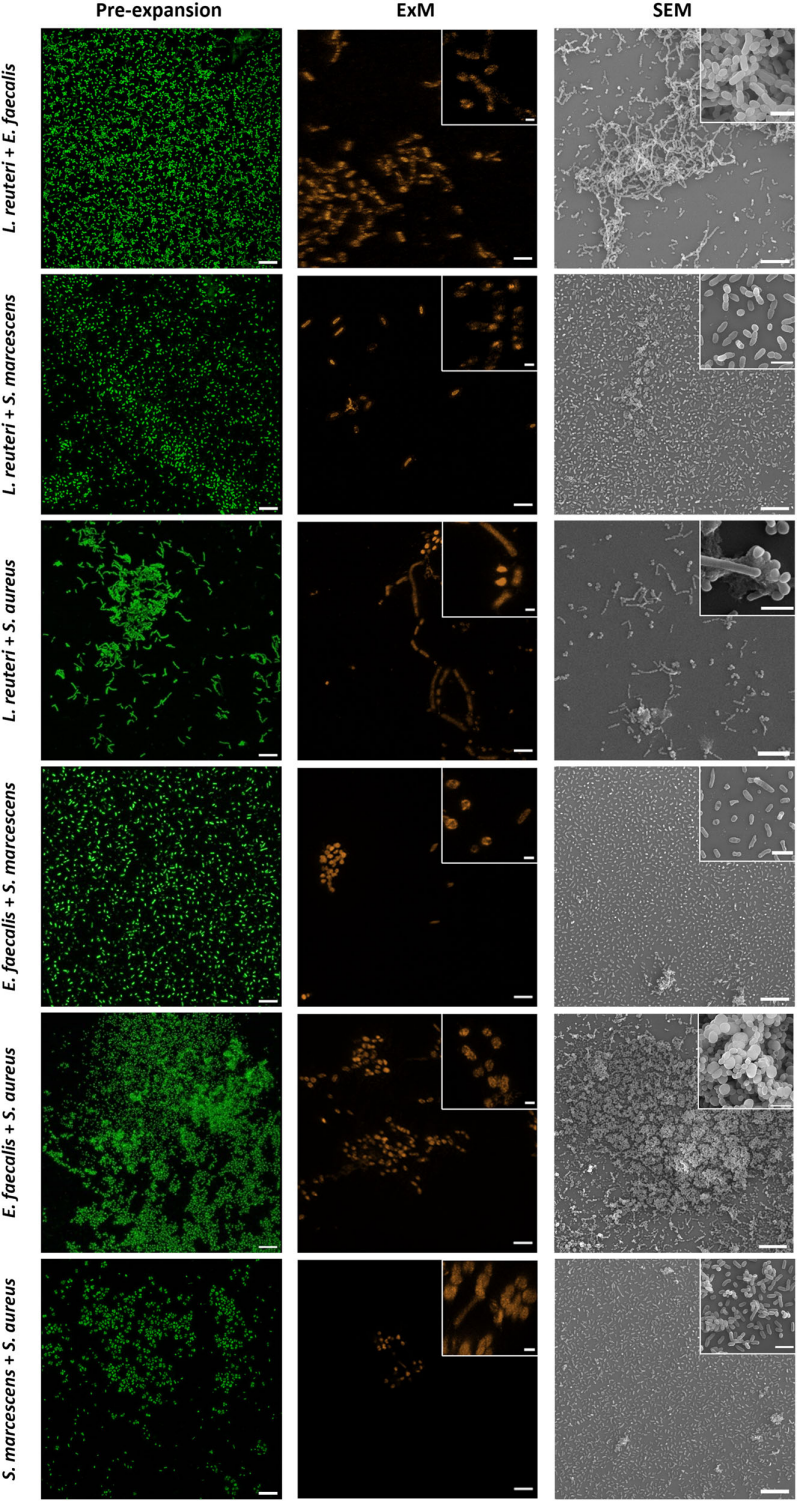
Representative images of dual-species biofilms (48 h) of *Limosilactobacillus reuteri*, *Enterococcus faecalis*, *Serratia marcescens* and *Staphylococcus aureus*. **Left** and **middle**. Pre-expanded and expanded biofilms as visualized by CLSM. Pre-expanded and expanded biofilms were labelled with SYTO 13 and Alexa Fluor 555 NHS ester, respectively. Scale bars: 10 μm. Inset scale bars: 2 μm. Expansion factors are 3.2 and 4.1 for *L. reuteri* + *E. faecalis*; 3.3 for *L. reuteri* + *S. marcescens*; 3.4 and 2.1 for *L. reuteri* + *S. aureus*; 2.55 and 2.1 for *E. faecalis* + *S. marcescens*; 2.8 for *E. faecalis* + *S. aureus* and 2.0 and 2.2 for *S. marcescens* + *S. aureus*. **Right**. Non-expanded biofilms as visualized by SEM. Magnifications are ×1000 (×5000, inset) for *L. reuteri* + *E. faecalis*, and *E. faecalis* + *S. marcescens;* ×500 (×9000, inset) for *E. faecalis* + *S. marcescens* and *S. marcescens* + *S. aureus* and ×1000 (×9000, inset) for *L. reuteri* + *S. aureus* and *E. faecalis* + *S. aureus*.

The combination *L. reuteri*-*E. faecalis* (BS1) did not alter the formation of the network pattern previously described for *L. reuteri* biofilms (Fig. 2). The presence of spherical shaped cells of *E. faecalis* was detected within the biofilm after expansion (Fig. 6). Interestingly, the species combination in BS2 lead to the formation of dispersed biofilms, where both *L. reuteri and S. marcescens* cells were indistinguishable (Fig. 6) mainly due to a shortening of *L. reuteri* cells^30^. *S. aureus* cells in BS3 induced the formation of a less condensed *L. reuteri* network decorated with small clusters containing *S. aureus* cells (Fig. 6). In the case of *E. faecalis* and *S. marcescens* biofilms, a rather sparse distribution of cells was found, but some clusters of *E. faecalis* cells were observed (Fig. 6). *E. faecalis* and *S. aureus* formed very dense biofilms containing a mixture of both species that were undistinguishable under ExM due to the coccus structure of both species (Fig. 6). Finally, BS6 was characterized by spread cells with small clusters enclosing both species (Fig. 6).

### The expansion factor in dual-species biofilms depends on bacterial combination and differs from their value in mono-species biofilms

**Fig. 7:**
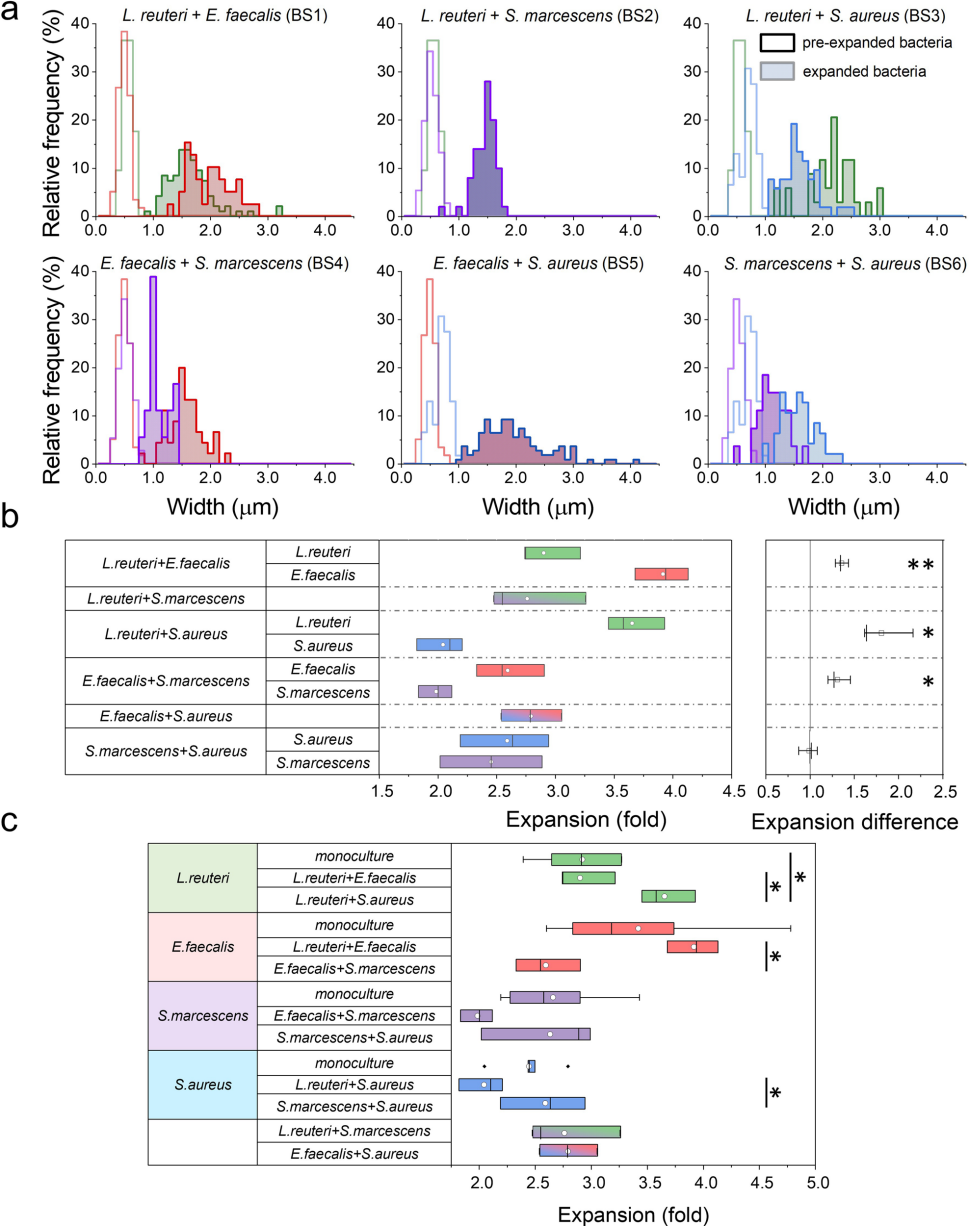
Expansion factors of dual-species biofilms. **a** Relative frequency of bacterial width of non-expanded (empty field) and expanded (coloured field) biofilms as measured from confocal images. *Limosilactobacillus reuteri* (green), *Enterococcus faecalis* (red), *Serratia marcescens* (violet) and *Staphylococcus aureus* (blue). **b** Expansion factors of dual-species biofilms. Average values ± standard deviation are: 2.9 ± 0.3 and 3.9 ± 0.2 (*n* = 3) for *L. reuteri* and *E. faecalis*, respectively in BS1; 2.7 ± 0.4 (*n* = 3) for both *L. reuteri* and *S. marcescens* in BS2 (species not distinguished); 3.6 ± 0.2 and 2.0 ± 0.2 (*n* = 3) for *L. reuteri* and *S. aureus*, respectively in BS3; 2.6 ± 0.3 and 2.0 ± 0.1 (*n* = 3) for *E. faecalis* and *S. marcescens*, respectively in BS4; 2.78 ± 0.3 (*n* = 3) for both *E. faecalis* and *S. aureus* in BS5 (species not distinguished); 2.6 ± 0.5 and 2.6 ± 0.4 (*n* = 3) for *S. marcescens* and *S. aureus*, respectively in BS6. **Right**. Note that *E. faecalis* has an enhanced expansion of 1.4 ± 0.1 (*n* = 3) and 1.3 ± 0.1 (*n* = 3) in BS1 and BS4 biofilms, respectively*. L. reuteri* shows an enhanced expansion of 1.8 ± 0.3 (*n* = 3) in BS3. BS6 does not present different expansions on their dual-species bacteria. Statistical significance: *(*p*-value < 0.05), **(*p*-value < 0.01); Tukey Mean Difference test). Boxplot elements are: white circle-mean; centre line-median; box limits-upper and lower quartiles; whiskers-extreme values. **c** Comparison between expansion factors measured in mono- and dual-species biofilms. *L. reuteri* expansion is significantly higher when growth with *S. aureus* or *S. marcescens*. Expansion of *E. faecalis* is significantly higher when growth with *L. reuteri* but lower when growth with *S. marcescens*. Expansion of *S. aureus* is significantly lower when growth with *L. reuteri*. Statistical significance: *(*p*-value < 0.05); Tukey Mean Difference test). Boxplot elements are: white circle-mean; centre line-median; box limits-upper and lower quartiles; whiskers-extreme values.

To assess how the presence of other bacterial species affects the expansion in dual-species biofilms we measured the linear expansion factor of individual strains within the same biofilm (Fig. 7a, b). Note that microbial species in BS2 and BS5 were indistinguishable as they shared very similar shape and dimensions (bacilli–bacilli and cocci–cocci, respectively). In these cases, both species were considered as one population. Remarkably, the linear expansion factor was not the same for different species within the same biofilm in BS1, BS3 and BS4 (Fig. 7b). *E. faecalis* presented an expansion factor 1.4 ± 0.1 and 1.3 ± 0.1 (*n* = 3) higher than their counterparts in BS1 (*L. reuteri*) and BS4 (*S. marcescens*), respectively. Also, *L. reuteri* expanded 1.8 ± 0.3 (*n* = 3) more than *S. aureus* in BS3. In contrast, *S. marcescens* and *S. aureus* exhibited similar expansion factors in BS6 (*n* = 3).

Additional information was obtained by comparing the linear expansion factors of different strains in BS with their intrinsic values found in mono-species biofilms (Fig. 7c). Again, the expansion factor was very dependent on the species combination and no correlation with particular conditions was found. *L. reuteri* expansion is significantly higher (*p* < 0.05) when forming dual-species biofilms with *S. aureus* (BS3) whereas it remains unaltered in presence of *E. faecalis* (BS2). Similarly, *E. faecalis* expansion is significantly higher (*p* < 0.05) when forming biofilms with *L. reuteri* (BS1) and remained similar when growing in the presence of *S. marcescens* (BS4). *S. aureus* expansion is significantly lower (*p* < 0.05) in the presence of *L. reuteri* (BS3) but similar in dual-species biofilms with *S. marcescens* (BS6). Finally, the expansions factors of *S. marcescens* were similar independently of the partner bacteria *(E. faecalis* or *S. aureus)*. All data are shown in Supplementary Tables 4, 5*.*

Furthermore, we observed that the expansion factor for a particular species changes when combined with different partners. In particular, *L. reuteri* expansion factor was on average ~3.5 when growing with *S. aureus* (BS3) and around 2.9 with *E. faecalis* (BS1) or alone. Similarly, *E. faecalis* expansion increased in the presence of *L. reuteri* (BS1) having similar values than in mono-species biofilms whereas the expansion factor of *E. faecalis* was reduced when forming dual-species biofilms with *S. marcescens* (BS4). Moreover, the association between *S. aureus* and *S. marcescens* (BS6) reduced the expansion ability of the *Enterobacteriaceae* compared with their mono-species biofilms.

### Detection of EPS matrix after expansion

**Fig. 8:**
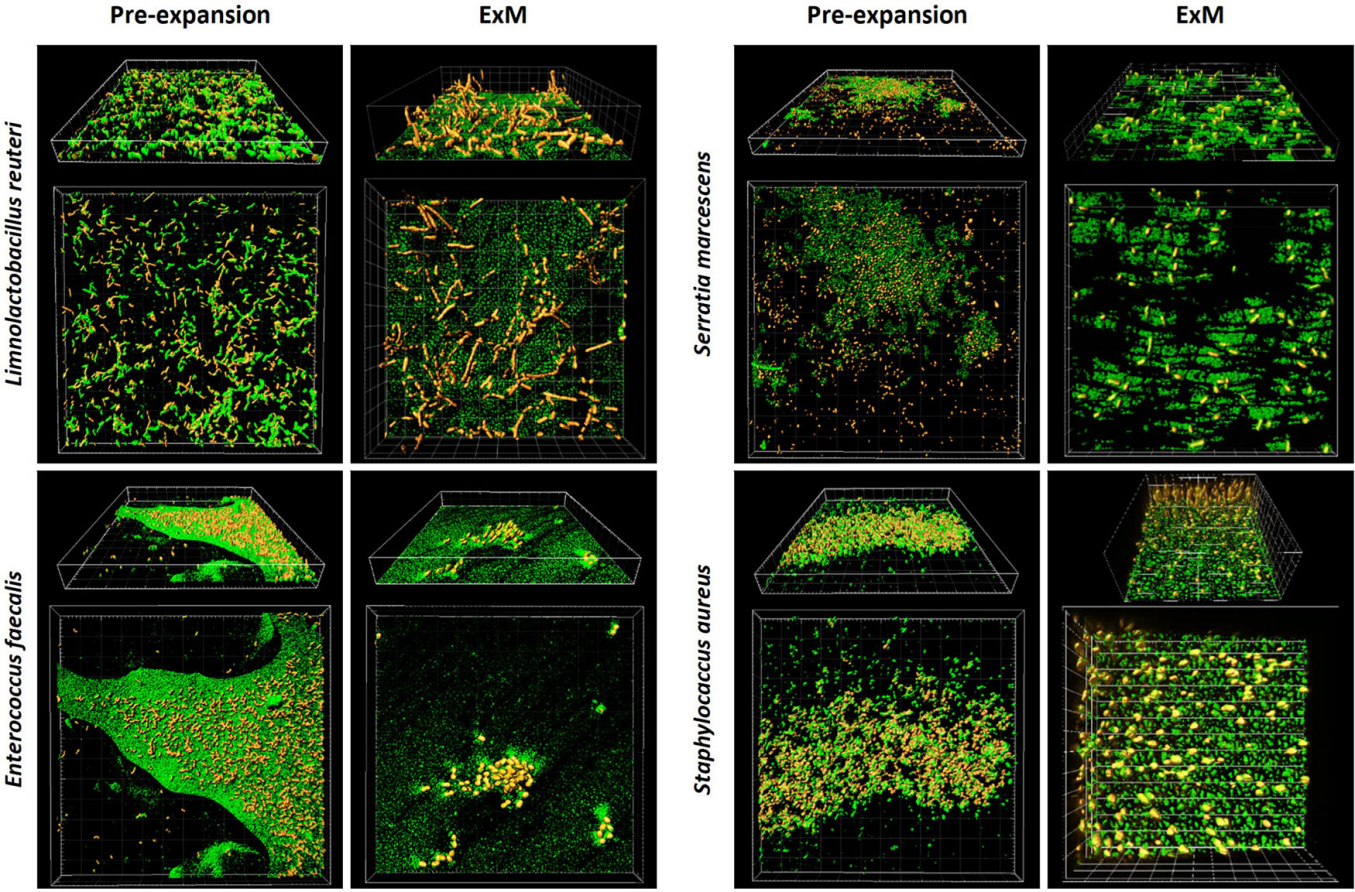
Detection of EPS matrix before and after expansion. Three-dimensional reconstitutions of pre-expanded and expanded mono-species biofilms (48 h) of *Limosilactobacillus reuteri*, *Enterococcus faecalis*, *Serratia marcescens* and *Staphylococcus aureus*. Proteome is labelled in red (Alexa Fluor 555 NHS ester) whereas the polysaccharide component of the EPS matrix is labelled in green (Concanavalin A–488). Grid square: 10 μm.

To further evaluate the EPS matrix integrity of biofilms after expansion, we labelled the polysaccharide and eDNA components using the specific stains Concanavalin A-488 and SYTOX Green, respectively. Whereas no difference between the DNA and the proteome channels was observed (Supplementary Fig. 5), the Concanavalin fluorescent bioconjugate revealed the EPS matrix surrounding bacteria in single-species biofilms (Fig. 8). Unlike non-expanded biofilms, the EPS matrix was not imaged homogeneously all along the expanded biofilm, suggesting that the ExM protocol may have partially damaged the biofilm structure. Similar results were obtained for dual-species biofilms (Supplementary Fig. 6).

**Fig. 9:**
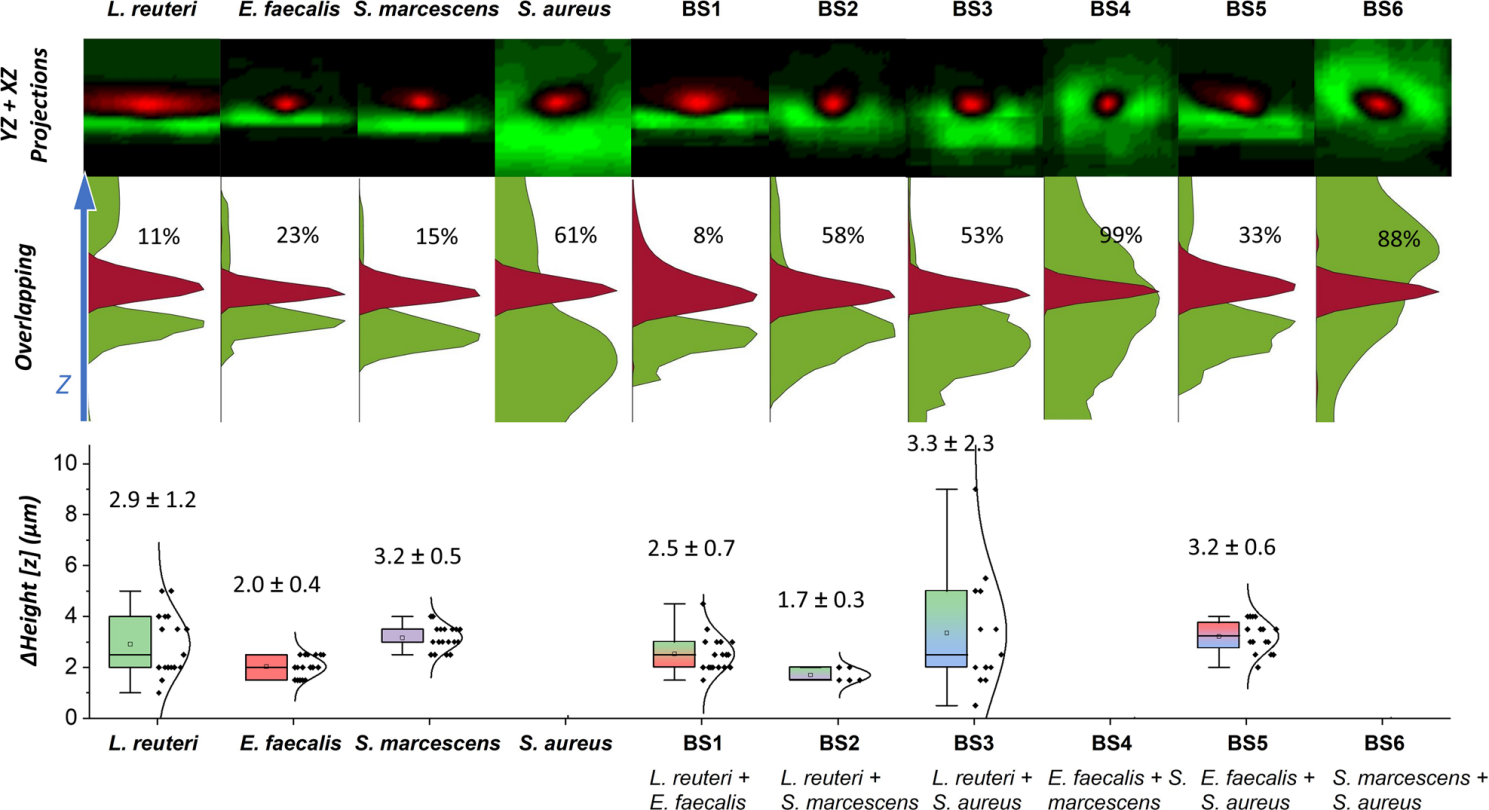
Relative *z*-position of single bacteria within the embedding matrix from mono- and dual-species biofilms after expansion. Cells were labelled with Alexa Fluor 555 NHS ester (red) and matrix was visualized by Concanavalin A-488 (green). **Top**. 2D reconstruction image from *YZ* + *XZ* projections from single bacterium (*n* = 10). For the reconstruction, images were centred using the brightest red fluorescence pixel. Dimensions of cropped images are 20 μm × 4.8 μm (vertical and horizontal directions). **Middle**. Fluorescence *z*-distribution of both the bacterial and matrix channels. An overlap above 60% is considered to describe a system with bacteria embedded into the biofilm matrix (*S. aureus*, BS4 and BS6) whereas below 60%, a carpet configuration is considered. *Z* arrow bar is 20 μm. For a better visualization, curves are normalized. **Bottom**. Boxplots of *ΔHeight*, defined as the difference between the maximum value for red fluorescence *z*-distribution and the maximum value for green fluorescence *z*-distribution. Average ± SD values are: *L. reuteri*: 2.9 ± 1.2 μm (*n* = 17); *E. faecalis*: 2.0 ± 0.4 μm (*n* = 17); *S. marcescens*: 3.2 ± 0.5 μm (*n* = 18); (BS1) *L. reuteri* + *E. faecalis*: 2.5 ± 0.7 μm (*n* = 18); (BS2) *L. reuteri* + *S. marcescens*: 1.7 ± 0.3 μm (*n* = 5); (BS3) *L. reuteri* + *S. aureus*: 3.3 ± 2.3 μm (*n* = 13) and (BS5) *E. faecalis* + *S. aureus*: 3.2 ± 0.6 μm (*n* = 16). Boxplot elements are black square-mean; centre line-median; box limits-upper and lower quartiles; whiskers- extreme values; diamonds-experimental data; curved line: normal distribution.

To evaluate bacteria localization within the EPS matrix we measured the fluorescence intensity *z*-distribution of both the bacterial and matrix channels from homogeneously expanded regions (Fig. 9, see Methods and Supplementary Fig. 7 for details). An overlap (>60%) of their respective distribution is taken as an indication of an embedding configuration whereas a carpet configuration was allocated when a shift between distributions was obtained. Whereas *L. reuteri*, *E. faecalis* and *S. marcescens* cells were found to grow over the matrix, *S. aureus* cells were embedded within the matrix. For dual-species biofilms, the embedding configuration was found in BS4 and BS6 biofilms whereas BS1-BS3 and BS5 biofilms were characterized by a carpet configuration (Fig. 9). The absence of the EPS stain by SYTOX Green provided us with a colocalization standard (Δ*z* = 0).

## Discussion

Unlike eukaryotic cell systems, bacterial specimens are still limited to ExM as they are protected by a cell wall that withstands pressure stress and prevents from lysis^31,32^. Cell wall consists of a peptidoglycan (PG) rigid layer composed of two sugar derivatives, N-acetylglucosamine and N-acetylmuramic acid. PG layer is primarily responsible for the strength of the wall and its chemical backbone is the same for all bacterial species. However, the PG proportion in the cell wall and the peptide chemistry differs from one species to another. Thus, a primary requirement for an efficient bacteria expansion is the breakdown of the cell wall. Furthermore, microorganisms are rarely isolated but associated forming biofilms in real environments. In these self-assembled structures, besides the inherent resistance of the bacterial cell wall, microorganisms are embedded into a polymeric matrix, which might represent a new physical barrier for ExM; acting as a diffusive barrier for digestion enzymes or preventing the mechanical expansion during expansion step.

In general, soft gels swell more than the stiff gels as they resist less mechanical deformation. Acrylamide-based hydrogels are widely used because their stiffness can be tuned over a wide range just by varying both the polymer and cross-linker concentrations, covering an extended variety of viscoelastic behaviour^33^. In our case, the rigidity of 10% acrylamide cross-linked with 0.25% of bisacrylamide would lead to the formation of a soft solid with a storage modulus of $G^{\prime}\sim 10\,{\rm{kPa}}$^33^. However, biofilms are characterized by a low mechanical rigidity, with typical shear moduli of $G^{\prime}\sim 10{\,\rm{Pa}}$^34^. The variability depends on the EPS composition, which self-adapts to environmental conditions^21^. In contrast, the mechanical rigidity of peptidoglycan is characterized by a storage modulus of $G^{\prime}\sim 10\,{\rm{MPa}}$ ^35^. Such a high value is required to prevent cells from bursting by the turgor pressure. Just by comparison of different rigidities, the swelling of acrylamide gels will concomitantly expands the extracellular matrix but it will be unable to expand cells with an undamaged cell wall^14^. This mechanical assessment underlines the enzymatic digestion of the cell wall as the main strategy for applying ExM to biofilms and preserving their structures as well as the spatial distribution of EPS components.

Enzymatic treatments targeting the cell wall are based in PG-targeting enzymes, alone or in combination with detergents. Lysozyme is the enzyme most widely used for this matter as it breaks the β-1,4-glycosidic bond between N-acetylglucosamine and N-acetylmuramic acid. Lysozyme digestion in combination with proteinase K had been used to successfully expand *Neisseria gonorrhoeae*, *Acetobacter tropicalis* and *Acidaminococcus intestine*^12^. So far, these studies have been done in bacterial suspensions of mono-species cultures. In this work we use as initial target for our experiments mono-species biofilms of both gram-negative (*S. marcescens*) and gram-positive (*L. reuteri, S. aur*e*us* and *E. faecalis*) strains isolated from clinical environments. In our systems, lysozyme digestion resulted in heterogeneous (non-isotropic) expansions especially in *L. reuteri* cells that exhibited aberrant morphologies after expansion (Supplementary Fig. 1). Note that the use of lysozyme had no effect on the expansion of *Lactiplantibacillus plantarum*^14^.

Next, we decided to use mutanolysin, another N-acetylmuramidase that has been successfully used for isotropic expansions of *L. plantarum*, *E. coli*, *A. tropicalis* and *A. intestini*^14^. Mutanolysin treatment was only effective for expansion of mono-species young (12 h) biofilms and an enzymatic digestion cocktail targeting not only the bacterial cell but also the matrix components was required to achieve an isotropic expansion of complex biofilms (*i.e*. 48 h and dual-species biofilms). Definitely, when we used a combination of both enzymes, mutanolysin and lysozyme, all the species in biofilms preserved their cell morphology and organization and expanded isotropically with an average expansion factor (Fig. 3c and Supplementary Table 2). Nevertheless, we found both linear and volumetric expansion factors were species-dependent (Fig. 3c and Supplementary Table 1). In previous expansion studies, such variability has been ascribed to bacterial cell wall dissimilarities between gram-positive and gram-negative microorganisms^14^. Whereas a 90% of the cell wall consists of PG in gram-positive bacteria, only about 10% of the cell wall is PG in gram-negative bacteria. Note that the behaviour of the three gram-positive bacteria was rather variable in our study.

Certainly*, S. aureus* biofilms expand significantly lower than those of *E. faecalis*. This observation may be due to a low efficiency of lysozyme and mutanolysin treatment to digest *S. aureus* cell wall. Alternatively, we ran additional ExM experiments to assess lysostaphin as a better digestion enzyme as it can strongly breaks down penta-glycine bridges in the peptidoglycans of *S. aureus* (Supplementary Fig. 8). The incubation with lysostaphin resulted into a similar expansion as that obtained with lysozyme and mutonolysin, with a linear expansion factor $F_{1D}^{ExM} \approx 2.1$. This experiment suggests that the biofilm matrix (both, quantity and quality) might be relevant for expansion in this case. As revealed by SEM images (Fig. 2) *S. aureus* biofilms produce a much denser polymeric matrix than the rest of the species. Moreover, biofilm matrix composition is variable^36^. Matrix components of *S. aureus* biofilms include proteins (some of them amyloids), polysaccharides such as poly-N-acetylglucosamine (PNAG), and eDNA^37–40^. These components can interact in complex ways leading to stronger interactions, such as those involving polysaccharides-eDNA and amyloids-eDNA^39,41^. Indeed, stiffer biofilms have been described for *S. aureus*, with a storage modulus up to $G^{\prime}\sim 1\,{\rm{kPa}}$^42^.

Although in most species polysaccharides are the major component of the extracellular matrix^43^, this is not the case for *E. faecalis*. In this microorganism, eDNA seems to play a pivotal role as matrix scaffold^44^. Indeed, the use of DNase I significantly affects its biofilm structure^45^. In our case, DNA labelling via SYTOX Green did not clearly reveal the presence of this component in the matrix. Only bacterial DNA, which co-localized with bacterial proteome, was observed (Supplementary Fig. 5). Although a better resolution might be achieved using anti-dsDNA fluorescence immunolabelling, we cannot discard that some reactions of the expansion protocol may have a detrimental effect upon this polymer or its abundance in the biofilm matrix of these strains was not enough for direct labelling and visualization. Thus, the higher expansion factor measured for *E. faecalis* in mono-species biofilms could be due to a loss of mechanical stiffness of its EPS support, where DNA could play a key role^34^. Besides, values of *S. marcescens* and *L. reuteri* expansion factors were intermediate to those achieved by *E. faecalis* and *S. aureus*. In the *Enterobacteriaceae* family, polysaccharides (mainly cellulose) and amyloids proteins are also involved in biofilm formation^46^. Similarly, the role of the exopolysaccharide levan (β-2,6-linked fructan) is essential for the gut colonization of *L. reuteri*^47^.

The simultaneous staining of EPS and bacteria (Fig. 8) suggests that expansion of both biofilm matrix and cells occur concomitantly, at least the polysaccharide part, as polysaccharide-labelling via Concanavalin A-488 revealed matrix engulfing microbial clusters after expansion (Fig. 8 and Supplementary Fig. 6). Moreover, visual inspection of biofilms before and after expansion shows that biofilm organization remains mostly conserved although some damage was observed; probably ascribed to the some reactions of the expansion protocol. More experiments are required to correlate the dissimilar values of the expansion factors obtained for different species with the chemical composition of the EPS and its possible alteration upon treatment. Expansion factors could be then regarded as a way of indirectly deciphering the mechanical and/or physical strength of the biofilm matrix as a function of the polysaccharide or DNA components.

When the same protocol was used for expanding dual-species biofilms the expansion factor of each species within the same system differs. This result suggests that the ecological relationships between the species within the same biofilm might be sustained through very different inter-species interactions. As the applied ExM protocol was the same for mono-species biofilms we cannot discard that species-interactions led to changes in the composition of the resulting biofilm matrix and thus in its physical properties. According to this, stronger inter-species interactions could be predicted for BS4 (*E. faecalis* and *S. marcescens*) and for BS6 (*S. aureus* and *S. marcescens*).

All the strains here used were isolated from the inner surface of nasogastric enteric tubes intended for feeding preterm children in a previous work^30^. In particular, *E. faecalis*, *S. marcescens* and *S. aureus* are three species commonly associated with outbreaks in neonatal intensive care units. Preterm children exhibit a broad clinical spectrum; including septicaemia, conjunctivitis, pneumonia, urinary tract infections, and meningitis and in the most severe of their manifestations they can even result in death^48–50^. The information provided by expanding biofilms could shed some light into the physical strength of inter-species interactions in these systems without the necessity of using more specialized techniques. More studies in multispecies biofilms are required to confirm these hypotheses.

In this work we propose for the first time to the best of our knowledge the use of ExM for studying mono- and dual-species biofilms. Taken together, our results demonstrate that the enzymatic digestion of cell wall in ExM preserves the morphology of embedding cells and maintain their relative arrangement within the EPS of the original biofilms (Fig. 4) while keeping the ability to resolve external cellular features at the nanoscale (Fig. 5). Although we present preliminary results for specific protein immunolabelling (Supplementary Fig. 4), its efficiency can be compromised by the denatured nature of proteins and the low penetration of antibodies. Indeed, the adapted protocol presented here combines physical denaturation steps^9^ with detergent-mediated permeation steps^14,25^ that might prevent the use of fluorescent fusion proteins for protein localization within the cell. The use of smaller nanobodies produced in denaturizing conditions remains as a possible way to improve the current protocol for a better detection and imaging of internal supramolecular structures at the nanoscale. Also, the implementation of fluorescent fusion proteins might be possible by changing the physical denaturation steps by an enzymatic-based treatment^13^. Additionally, the combination of our protocol with click-ExM^10^ could be applied for studying matrix components and their interactions, providing valuable information for the development of more effective treatments for biofilm removal.

## Methods

### Bacterial strains and growth conditions

Two lactic acid bacteria (LAB), *Limosilactobacillus reuteri* 7SNG3 and *Enterococcus faecalis* 15SNG3, and three nosocomial pathogens, *Serratia marcescens* 10SNG3, *Staphylococcus aureus* 45SNG3 and *Escherichia coli* 8H3-10 strains were used as model microorganisms for this work. All of them were isolated in a previous work from the inner surface of nasogastric enteral feeding tubes inserted for 48 h into preterm infants and later on identified by sequencing of 16 S rRNA gene^30^. An isolated colony of each strain was transferred into a tube with 10 mL of Brain Heart Infusion (BHI, Oxoid; Basingstoke, UK) and incubated aerobically overnight at 37 °C. Cells were recovered after washing twice by centrifugation at 17,000 *× g* for 10 min at 4 °C and suspended into 10 mL of the same culture medium. The optical density of the bacterial suspensions at 600 nm (OD_600_) was adjusted to 0.1 (~10^7^ cfu/mL).

### Experimental system for biofilm development

Biofilms were developed in a batch system^51^. In this, thirty-two microscope circular coverslips (10 mm diameter Ø) (Thermo Scientific, Germany) were used as adhesion substrate. They were inserted vertically into the narrow radial slits of a Teflon platform (6.6 cm diameter). An axial stainless steel rod was used for handling the platform and its lid into a 600 mL glass beaker and was heat-sterilized as unit. 40 mL of BHI were inoculated with 600 μL of the previously obtained cellular suspensions (10^7^ cfu/mL) to obtain a starting concentration of 10^5^ cfu/mL. For dual-species biofilms both strains were inoculated in 1:1 proportion. These systems were incubated aerobically at 37 °C for 48 h. Control biofilm samples were pre-fixed (see *Biofilm fixation* section) and labelled with SYTO 13 for confocal imaging without expansion (see *Image acquisition* section).

### Biofilm fixation and expansion reagents

Ethanol, PBS, sodium chloride, potassium chloride, sodium phosphate dibasic heptahydrate, potassium phosphate monobasic, triton X-100, tween 20, lysozyme, paraformaldehyde (PFA), glutaraldehyde (GA), acrylamide (AAm) solution, N,N′-(1,2-Dihydroxyethylene) bisacrylamide (DHEBA), sodium acrylate (SA), sodium dodecyl sulfate and tris(hydroxymethyl)aminomethane (Tris) were purchased from Sigma-Aldrich. 6-((acryloyl)amino)hexanoic Acid, Succinimidyl Ester (Acryloyl-X SE), TEMED, APS and mutanolysin were supplied by Thermo Fisher.

### Biofilm fixation

Cultured biofilms were pre-fixed with ethanol 70–75% for 1 h at −20 °C^25^. Pre-fixed samples could remain up to two weeks at −20 °C until further fixation treatment. After pre-fixation, samples were rinsed with phosphate buffer saline (PBS) (137 mM NaCl, 2.7 mM KCl, 10 mM Na_2_HPO_4_, and 1.8 mM KH_2_PO_4_) for 10 min at room temperature and treated with Triton X-100 0.3% in PBS for 1 h on a rocking platform at room temperature for bacterial permeation^14,25^. Permeabilized biofilms were then digested overnight on a rocking platform at 37 °C with 160 U/mL mutanolysin for 12 h mono-species biofilms and 160 U/mL mutanolysin with 5 kU/mL lysozyme for mature (48 h) mono- and dual-species biofilms. Additionally, 160 U/mL mutanolysin and 640 U/mL mutanolysin with 20 kU/mL lysozyme digestion on mature mono-species biofilms were tested (see Supplementary Figs. 1, 2). After digestion, samples were treated as pan-ExM protocol^9^. Biofilms were fixed with 3% PFA + 0.1% GA in PBS 15 min at room temperature, and washed with PBS for 10 min. Next, biofilms were post-fixed with 1% AAm, 0.7% PFA, 100 µM Acyloyl-X SE for 3 h on a rocking platform at room temperature (Fig. 1) and washed twice with PBS for 10 min. Post-fixed samples could remain up to two weeks on ethanol 70–75% at −20 °C until expansion treatment.

### Expansion gelation chamber

The gelation chamber was built following ref. ^9^. Briefly, a ~150 µm thick chamber was built using two 22 × 22 mm cover slips (#1, Eprendia) as spacers placed over the sides of a third 60 × 22 mm coverslip (#1, Eprendia). After the addition of gel solution, a fourth cover slip is placed on top for sealing the chamber.

### Biofilms expansion

Fixed biofilms were located on the gelation chamber, and embedded in the expansion gel solution (10% AAm, 0.1% DHEBA, 19% SA, 0.25% TEMED, 0.25% APS)^9^. Samples gelled for 10–15 min at room temperature and incubated at 37 °C on a wet chamber for 1.5 h. Hydrogels were then incubated in 10 mL denaturation buffer (200 mM SDS, 200 mM NaCl, 50 mM Tris in H_2_O, pH 6.8) and warmed up to 45–50 °C^9^ on petri dishes for 15–30 min on a rocking platform at room temperature. Detached gels were then cut and transferred to a 1.5 or 2 mL Eppendorf filled with 1 mL of denaturation buffer and incubated at 76 °C for 1 h for sample homogenization (Fig. 1). Gels were then washed twice with PBS for 20 min each, borders were cut, and gels were stored at 4 °C on PBS. After homogenization gels were labelled (see below) and expanded. Labelled gels were embedded three times on fresh H_2_O over 30 min each on a rocking platform at room temperature for expansion (Fig. 1). Expanded gels were stored at 4 °C for image acquisition.

### NHS ester biofilm labelling

Pre-expanded gels were incubated with 20 µg/ µL Alexa Fluor 555 NHS ester or Alexa Fluor 488 NHS ester (ThermoFisher) dissolved on 100 mM sodium bicarbonate solution (Sigma-Aldrich) pH 8.4 on a rocking platform at room temperature^9^. Gels were subsequently washed twice in PBS supplemented with Tween 20 (0.1%) (PBS-T) for 20 min on a rocking platform at room temperature.

### Nucleic acids labelling

After NHS labelling, gels were incubated with SYTOX Green (invitrogen) in HBSS calcium- and magnesium-free buffer (NaCl 100 mM, KCl 5 mM, Na_2_HPO_4_ 0.3 mM, KH_2_PO_4_ 0.4 mM, Glucose 6 mM, NaHCO_3_ 4 mM) for 30 min on a rocking platform at room temperature^9^. Gels were subsequently washed twice with PBS-T for 20 min on a rocking platform at room temperature.

### FtsZ inmunolabelling in *E. coli* biofilms

Pre-expanded gels were incubated with the anti-FtsZ polyclonal antiserum MVC2 diluted 1:500 in antibody dilution buffer (0.2% BSA, 0.05% Tween 20, PBS 1X). The primary antibody was incubated overnight (~16 h) at 4 °C. Gels were then washed with 0.2% BSA, 0.1% Tween 20, PBS 1X twice for 20 min on a rocking platform at RT. Next, gels were incubated with Alexa555-conjugated anti-rabbit antibody (Sigma-Aldrich) diluted 1:500 in antibody dilution buffer for 5 h at 37 °C. Gels were subsequently washed twice with PBS-T 10 min and continue to NHS ester labelling.

### Matrix biofilm labelling

After NHS labelling, gels were incubated with 200 µg/mL Concanavalin A-Alexa Fluor 488 conjugated (ThermoFisher) dissolved on 2 mM sodium azide (Sigma-Aldrich) on a rocking platform at room temperature. Gels were subsequently washed twice in PBS-T for 20 min on a rocking platform at room temperature.

### Image acquisition and processing

Confocal images were obtained using a Nikon Ti-E inverted microscope equipped with a Nikon C2 confocal scanning confocal module, 488-nm and 561-nm continuous lasers (Sapphire), Plan Apo 100× NA 1.45 oil immersion objective (Nikon). *Z*-stack images of 0.5 μm *z*-step were taken for every sample. Three-dimensional projections from *z*-stacks were carried out using FIJI software. Three-dimensional reconstitutions from *z*-stacks were performed with IMARIS 8.0 software (Bitplane, Zürich, Switzerland).

### Biofilm expansion factor calculation

Expansion factors (${{{\mathrm{F}}}}^{{{{\mathrm{ExM}}}}}$) were calculated using two independent methods. For the first method, the width of single cells was measured directly with FIJI software on pre-expanded and expanded biofilms (Figs. 3a, b and 6a). Note that width of cells can be measured precisely regardless of cellular orientation in three dimensions^14^. The expansion factor for each species was then computed as the ratio between the widths of cells measured in expanded and pre-expanded biofilms; ${{{\mathrm{F}}}}_{1{{{\mathrm{D}}}}}^{{{{\mathrm{ExM}}}}} = {{{\mathrm{width}}}}_{expanded}/{{{\mathrm{width}}}}_{{{{\mathrm{pre}}}} - {{{\mathrm{expansion}}}}}$. For the second method, three-dimensional reconstitutions from *z*-stacks were obtained with IMARIS 8.0 software (Bitplane, Zürich, Switzerland) and the volume of single cells were obtained for pre-expanded and expanded biofilms. For each species, volumes followed skewed distributions, where their maxima were obtained after fitting to LogNormal functions (Supplementary Fig. 3 and Supplementary Table 1). As volume expansion scales with a λ^3^ factor^24^, the volumetric expansion factor was then quantified as the cube root of the ratio between the volume of cells measured in expanded and pre-expanded biofilms; ${{{\mathrm{F}}}}_{3{{{\mathrm{D}}}}}^{{{{\mathrm{ExM}}}}} = \root {3} \of {{{{{\mathrm{V}}}}_{expanded}/{{{\mathrm{V}}}}_{pre - expansion}}}$. Expansions were considered isotropic when ${{{\mathrm{F}}}}_{1{{{\mathrm{D}}}}}^{{{{\mathrm{ExM}}}}} \approx {{{\mathrm{F}}}}_{3{{{\mathrm{D}}}}}^{{{{\mathrm{ExM}}}}}$.

### Determination of the spatial distortion of biofilms upon expansion

To analyze the spatial distortion resulting from expansion, the following procedures were implemented. Regions of interest in post-expanded images were initially aligned with pre-expanded images through a rigid transformation using the FIJI TurboReg plugin. For a more refined alignment, two sequential non-rigid registrations were executed, using an implemented sequential affine and B-spline-based *MATLAB* algorithm^52^. Briefly, post-expanded images were Gaussian filtered (6-pixel radius) and subsequently binned to align with the pre-expansion image of the same field of view. For the B-spline registration, a penalty registration of 1e^−3^ was applied^6,9^. From the deformation vector field obtained through the B-spline registration, the root mean square (RMS) error of expansion was computed across 10 μm distance measurements. The RMS error was calculated as the absolute difference between the distance measurements of each pair of points in the pre-expansion image ($a_{ij}^{pre}$) and their corresponding deformed coordinates ($a_{ij}^{def}$). A total of 10 fields of view were examined from a single expansion experiment.

### Matrix organization

To visualize the extracellular matrix after expansion, a dual colour stain was performed in mono- and dual-species biofilms. The protein channel was revealed through Alexa Fluor 555 NHS ester and the localization of the EPS matrix or the eDNA component was visualized with Concanavalin A-488 or SYTOX Green, respectively. As SYTOX Green only detected the nuclei acids within cells, the co-localization of both the protein and DNA channels was considered as a Δ*z* reference for the average position of cells within the embedding matrix. To determine the relative position of cells within the EPS matrix (Fig. 9 Top), crops of single cells were analyzed to obtain the maximum accumulative intensities on *Z* for both *XZ* and *XY* projections on the red (Alexa 555) and green (Concanavalin A-488) channels using a *MATLAB* homemade algorithm (see Supplementary Fig. 7 for details). The normalized fluorescence distributions for both channels were then plotted and the percentage of overlap between the red and green signals was calculated (Fig. 9 Middle). Overlapping below 60% was considered as a carpet configuration, whereas an overlapping above 60% was considered as an embedding configuration. For carpet configuration, distances between bacteria and EPS matrix were plotted using boxplots, including the main value and standard deviation (Fig. 9 Bottom).

### Visualization of biofilms by scanning electron microscopy

SEM images of the biofilms were acquired at the facilities of the National Centre of Electronic Microscopy (Complutense University of Madrid, Spain). Firstly, coverslips were extracted from the carousel platform, washed in 50 mL of sterile saline solution 0.85% (w/v) and then fixed by immersion into a solution containing 4% parafolmaldehyde (Sigma Aldrich, Spain) and 3% glutaraldehyde (Sigma Aldrich, Spain) in 0.1 PBS pH 7.2 for 12 h at 4 °C. Then, samples were rinsed with MilliQ water and dehydrated progressively by passage through a graded series of ethanol solutions ranging from 40% (v/v) to 100% (v/v). Critical point dehydration of the samples was carried out in a Leica CPD300 (Leica, Germany) using carbon dioxide as the transition fluid and coated with gold palladium in an automated sputter coater Leica EM ACE200 (Leica, Germany). Biofilms were visualized using a JEOL 6400 JSM electron microscope.

### Statistics

Expansion factors and isotropy for mono- and dual-species were tested via ANOVA. Differences between means were assessed using Tukey Mean Difference test. Each species of pair mixed biofilms difference expansion was tested through *t* Student analysis (mean > 1). Statistical significance difference was established as ns (*p*-value > 0.05), *(*p* ≤ 0.05) or **(*p* ≤ 0.01).

### Reporting summary

Further information on research design is available in the Nature Research Reporting Summary linked to this article.

### Supplementary information

Supplemental Material

Reporting Summary

## Data Availability

The authors declare that the data supporting the findings of this study are available within the paper and its Supplementary Information files. Should any raw data files be needed in another format they are available from the corresponding author upon reasonable request.
