# Supplementary material for: Expansion microscopy applied to mono- and dual-species biofilms
Source: NPJ Biofilms Microbiomes. 2023 Dec 5;9:92. doi: 10.1038/s41522-023-00460-x (PMC10696089; doi:10.1038/s41522-023-00460-x)
Supplement: Supplementary file 1 — Supplemental Material [file 41522_2023_460_MOESM1_ESM.docx]

**Supplementary Information**

**Expansion microscopy applied to mono- and dual-species biofilms**

David Valdivieso González^1,2,3^, Josué Jara ^4^, Víctor G. Almendro-Vedia^2,3,5^, Belén Orgaz^5^ and Iván López-Montero^1,2,3,*^

*^1^Dto. Química Física, Universidad Complutense de Madrid, Avda. Complutense s/n 28040 Madrid, Spain*

*^2^Instituto Pluridisciplinar, Universidad Complutense de Madrid, Ps. Juan XXIII 1, 28040 Madrid, Spain*

*^3^Instituto de Investigación Biomédica Hospital Doce de Octubre (Imas12), Avda. de Córdoba s/n, 28041 Madrid, Spain*

*^4^Sección Departamental de Nutrición y Ciencia de los Alimentos, Facultad de Veterinaria, Universidad Complutense de Madrid, 28040, Madrid, Spain*

*^5^Sección Departamental Farmacia Galénica y Tecnología Alimentaria, Facultad de Veterinaria, Universidad Complutense de Madrid, Avda. Complutense s/n 28040 Madrid, Spain*

***Corresponding author**: [ivanlopez@quim.ucm.es](mailto:ivanlopez@quim.ucm.es)

**Keywords:** Expansion microscopy, biofilms, *Staphylococcus aureus*, *Limosilactobacillus reuteri*, *Serratia marcescens*, *Enterococcus faecalis*, *Escherichia coli*, nosocomial pathogens, bacteria.


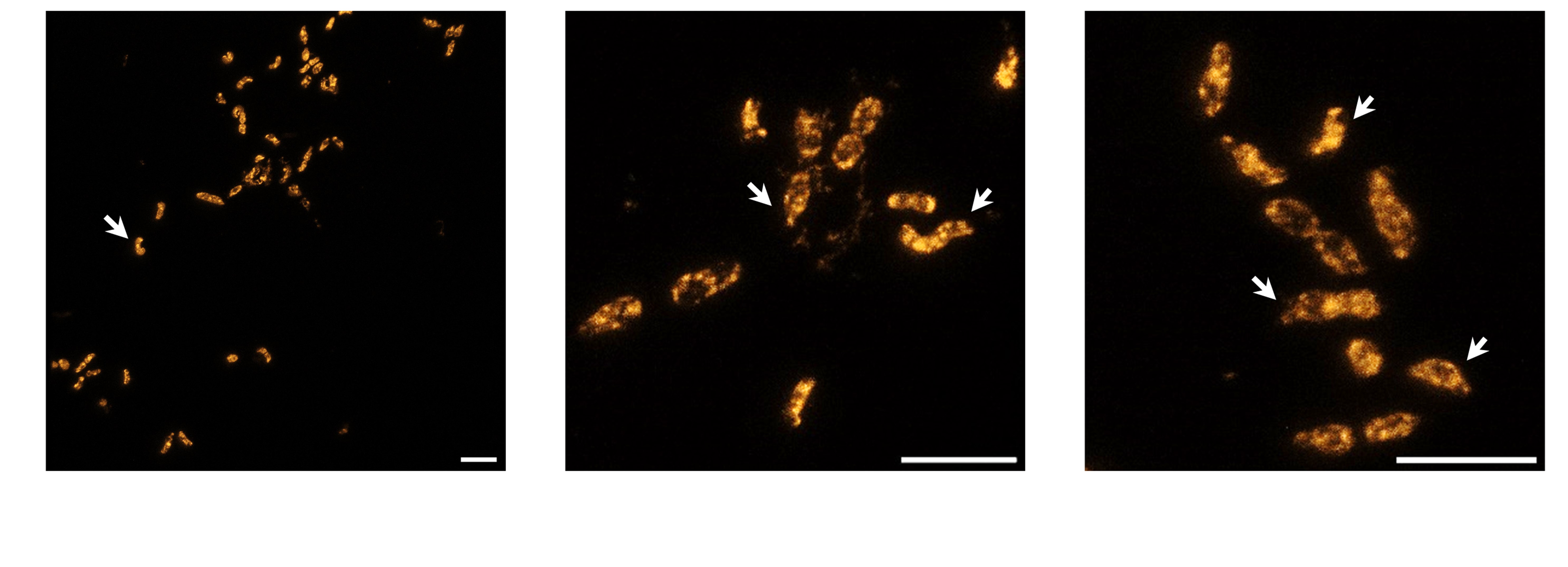


**Supplementary Figure 1.** **Representative images of *Limosilactobacillus reuteri* biofilms (12h) digested only with lysozyme.** White arrows point bacterial bumps and abnormal morphologies due to anisotropic expansion of partial digested biofilms. Scale bars: 10 μm.

**
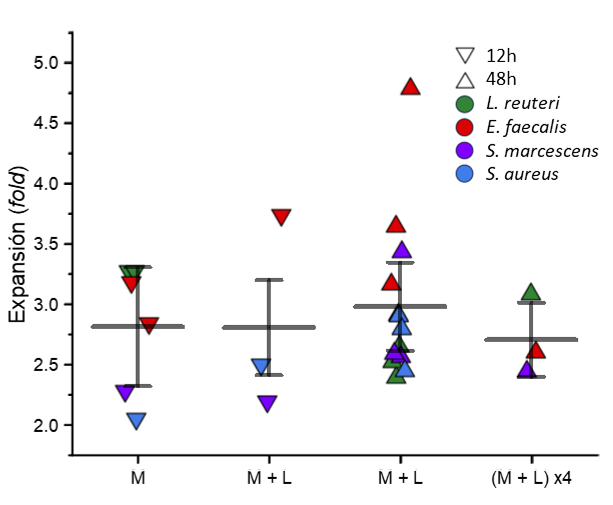
**

**Supplementary Figure 2. Expansion factors calculated for biofilms (12h or 48h) treated with different digestion conditions.** M, 160 U/mL mutanolysin; M + L, 160 U/mL mutanolysin and 5 kU/mL lysozyme; (M+L) ×4; 640 U/mL mutanolysin and 20 kU/mL lysozyme. Error bars correspond to SD.

**Supplementary Figure 3. Volume distributions of pre-expanded and expanded mono-species biofilms.** Volumes of single cells were calculated from *z*-stack images using IMARIS software. Each volume was obtained using IMARIS with a background subtraction parameter of 2.00 μm. The volume distributions were fitted to a LogNormal function (straight curves). In some cases, distributions were fitted to a bimodal function (yellow) including higher volumes, which correspond to two or more cells in such a close proximity that image processing considered as a single object. For this reason, only the maximum value of distributions was used as the average values with an estimated error of ± half of the bin size. Data are reported in Supplementary Table 3.


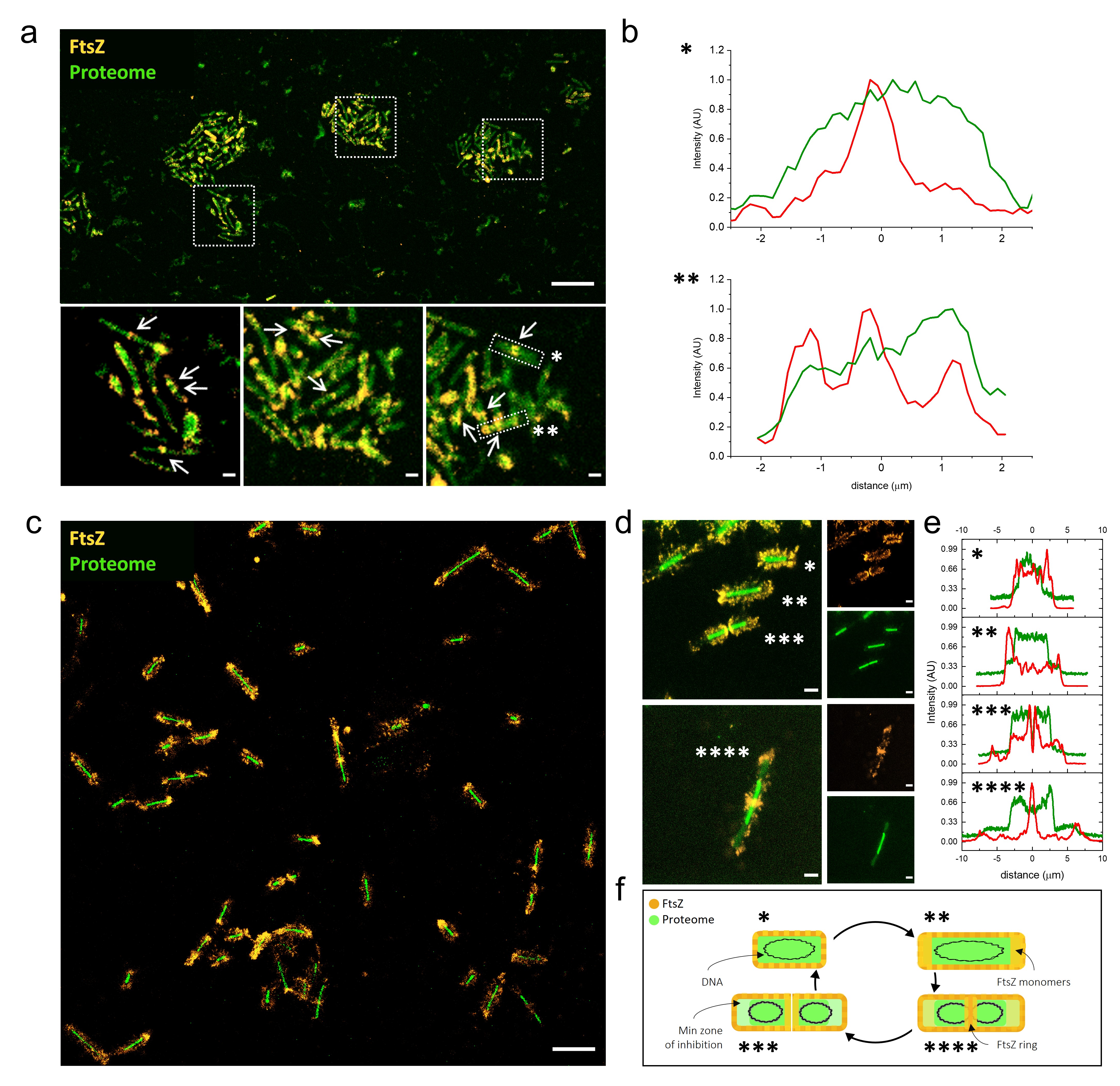


**Supplementary Figure 4. Fluorescence immunolabelling of FtsZ in pre-expanded and expanded biofilms of *E. coli*. A)** Representative image of a pre-expanded biofilm of *E.coli* as visualized by CLSM. Proteome was labelled with Alexa Fluor 488 NHS (green channel) and FtsZ was labelled with an anti-FtsZ polyclonal antiserum and a secondary Alexa555-conjugated anti-rabbit antibody (red channel). Scale bar is 10 μm. **Bottom:** ROIs from dashed squares in the large field of view. White arrows indicate FtsZ rings. Scale bars: 1μm. **B)** Intensity profiles from framed bacteria (**a** and **b**) in the bottom right image. **C)** Representative image of an expanded biofilm of *E.coli* as visualized by CLSM. Proteome was labelled with Alexa Fluor 488 NHS (green channel) and FtsZ was labelled with an anti-FtsZ polyclonal antiserum and a secondary Alexa555-conjugated anti-rabbit antibody (red channel). Scale bar is 10 μm. Expansion factor is 4.1. **D)** ExM images of *E. coli* bacteria in a biofilm. Images are representative for different states of division and FtsZ localization. **E)** Intensity profiles from selected bacteria (**a**, **b**, **c** and **d**) shown in D). **F**) FtsZ localization within *E. coli* during the cell division cycle.

**
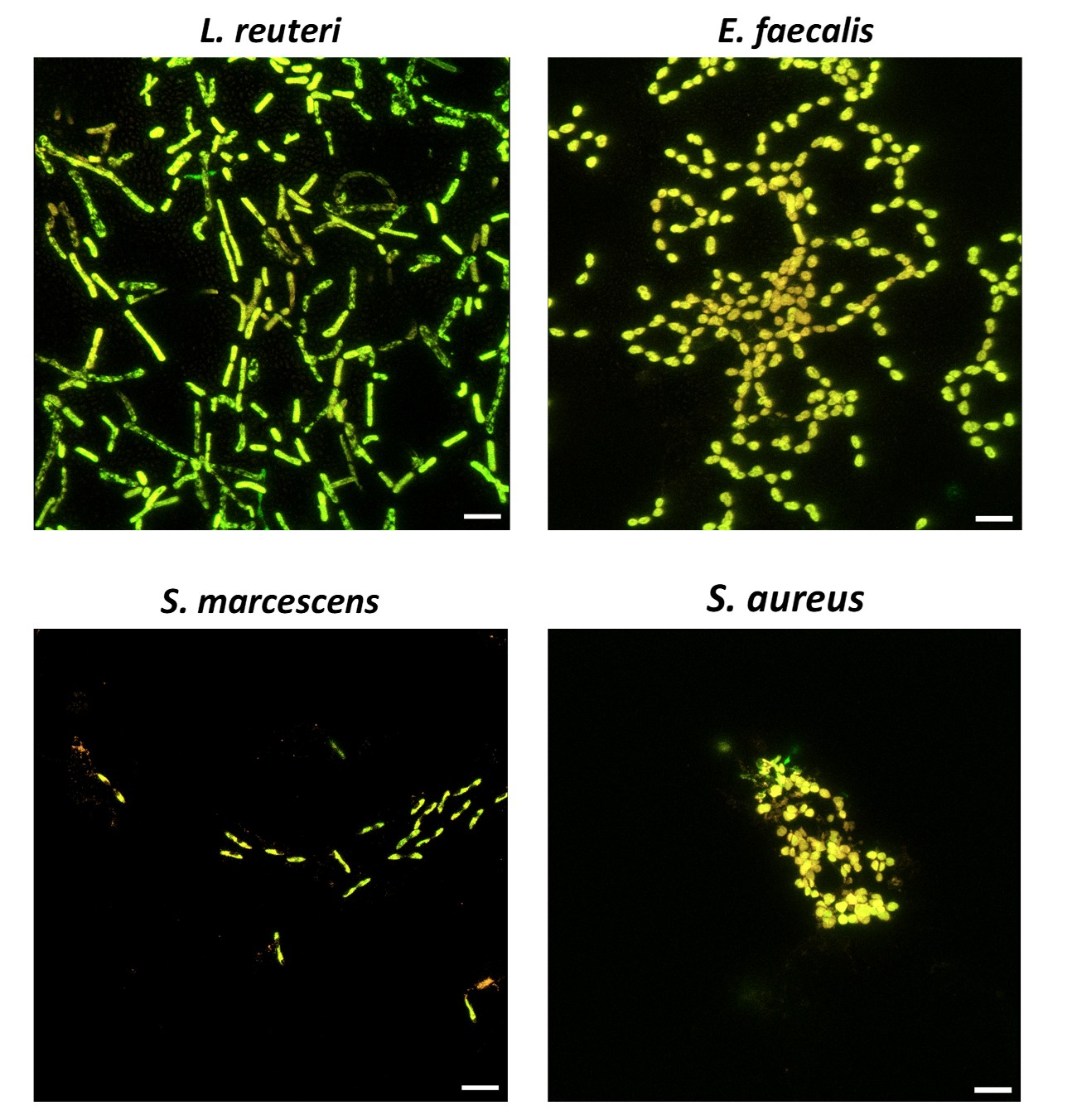
**

**Supplementary Figure 5. DNA labelling in Representative images of mono-species biofilms (48h) of *Limosilactobacillus reuteri*, *Enterococcus* *faecalis*, *Serratia* *marcescens* and *Staphylococcus* *aureus*.** Proteome is labelled in red (Alexa 555) whereas DNA is labelled in green (SYTOX Green). Bacterial DNA and proteome colocalize whereas eDNA is not stained. Scale bars: 10 μm.

**
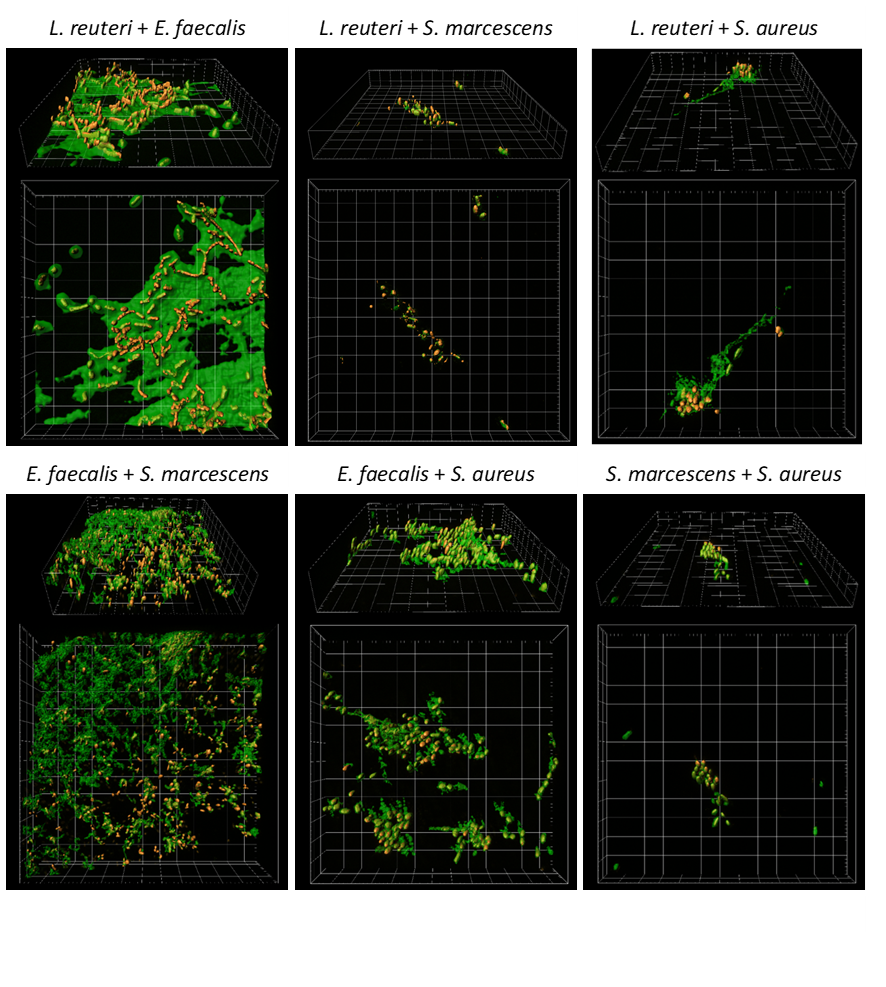
**

**Supplementary Figure 6. Three-dimensional reconstitutions of expanded dual-species biofilms (48h) of *Limosilactobacillus reuteri*, *Enterococcus* *faecalis*, *Serratia* *marcescens* and *Staphylococcus* *aureus.*** Proteome is labelled in red (Alexa 555) whereas the polysaccharide component of the EPS matrix is labelled in green (Concanavalin A–488). Grid square: 10 μm.

*
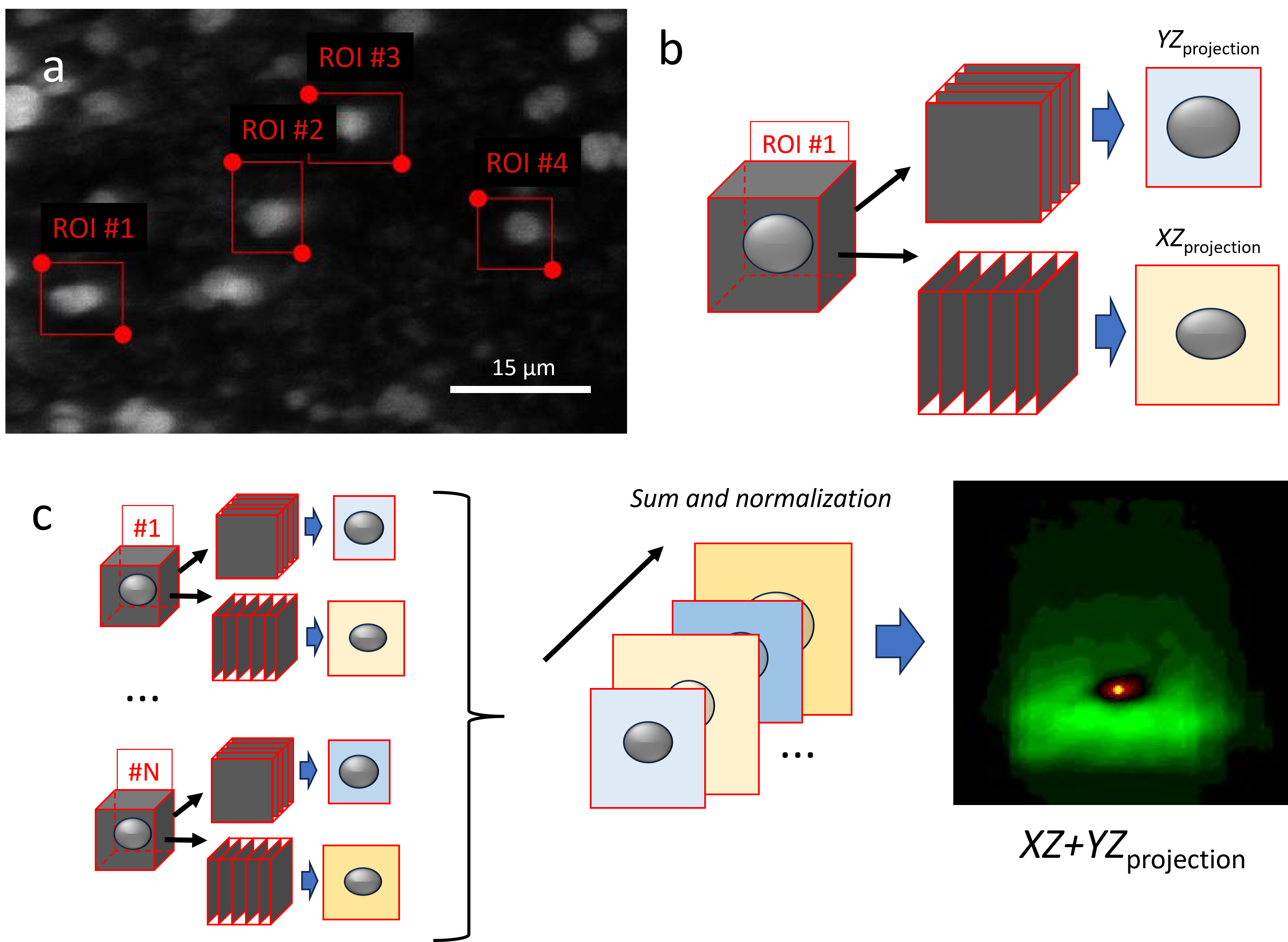
*

**Supplementary Figure 7. Procedure for calculating the *YZ* + *XZ* projection of embedded cells into the extracelular matrix. A)** Field of view of a 3D stack where several ROIs have been selected for further analysis. The cubic boxes contain the fluorescence intensity values of single pixels in *XYZ* from both cell (red) and matrix (green) channels. **B)** Projections *XZ* and *YZ* are built by addition of fluorescence intensity from all *XZ* and *YZ* pixels, respectively. **C)** Assuming a spherical symmetry, the *XZ* + *YZ* projection iresults form the combination of the *XZ* + *YZ* projection. The procedure is repeated for different cells to obtain the averaged and normalized *XZ* + *YZ* projection. For an optimal averaging among different planes and bacteria, ROIs are centred using the highest fluorescence signal from single cells. The same protocol is applied to different species (see **Figure 9** in the main text).

**
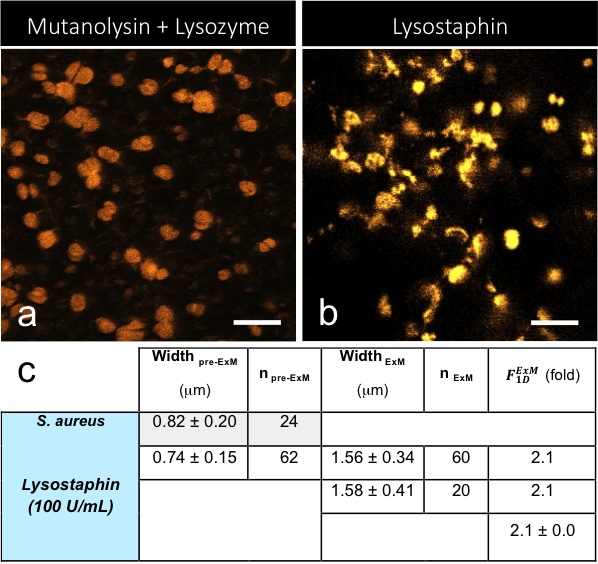
**

**Supplementary Figure 8. Representative images of expanded mono-species biofilms (48 h) of *Staphylococcus* *aureus*** using **A)** the combined action of mutanolysin and lysozyme and **B)** lysistaphin. Expanded biofilms were labelled with Alexa Fluor 555 NHS ester. Scale bars: 10 μm. **C)** Widths and expansion factors of *Staphylococcus aureus* in mono-species biofilms. Values of Widths and $\boldsymbol{F}_{\boldsymbol{1}\boldsymbol{D}}^{\boldsymbol{ExM}}$represent mean average ± standard deviation. Data of pre-ExM in grey cells were obtained from SEM micrographs as reference.

**Supplementary Table 1.** Widths, volumes (V) and Expansion Factors ($\mathbf{F}^{\mathbf{ExM}}$) obtained by two independent methods: (1) directly measuring the width of single cells from pre-expanded and expanded biofilms using SEM ($\mathbf{F}_{\mathbf{1D}}^{\mathbf{ExM}}$) and (2) from the cubic root of the ratio between the average volume of the cells before and after expansion using IMARIS ($\mathbf{F}_{\mathbf{3D}}^{\mathbf{ExM}}$). Values represent mean average ± standard deviation.

|  | **SEM Width (μm)** | **SEM Length  (μm)** | **V_Theory_ (μm)** | **V_Pre-Expansion_ (μm^3^)** | **V_Expanded_ (μm^3^)** | $\mathbf{F}_{\mathbf{3D}}^{\mathbf{ExM}}$ **(fold)** | $\mathbf{F}_{\mathbf{1D}}^{\mathbf{ExM}}$ **(fold)** |
| --- | --- | --- | --- | --- | --- | --- | --- |
| ***L. reuteri*** | 0.65 ± 0.06 | 1.50 ± 0.10 | 1.99 ± 0.18 | 2.03 ± 0.15 | 52.33 ± 5.00 | 2.95 ± 0.19 | 3.0 ± 0.3 |
| ***E. faecalis*** | 0.60 ± 0.09 | - | 0.90 ± 0.27 | 1.37 ± 0.10 | 22.04 ± 2.00 | 2.52 ± 0.03 | 3.4 ± 0.7 |
| ***S. marcescens*** | 0.50 ± 0.04 | 1.20 ± 0.10 | 0.94 ± 0.12 | 0.89 ± 0.10 | 25.62 ± 1.50 | 3.06 ± 0.08 | 2.6 ± 0.4 |
| ***S. aureus*** | 0.80 ± 0.15 | - | 2.14 ± 0.45 | 1.81 ± 0.25 | 22.58 ± 2.50 | 2.31 ± 0.15 | 2.4 ± 0.3 |

**Supplementary Table 2**. Widths and expansion factors of *Limosilactobacillus reuteri*, *Enterococcus faecalis*, *Serratia marcescens* and *Staphylococcus aureus* in mono-species biofilms. Values of Widths and $\boldsymbol{F}_{\boldsymbol{1}\boldsymbol{D}}^{\boldsymbol{ExM}}$represent mean average ± standard deviation.

|  | **Width _pre-ExM_**  (μm) | **n _pre-ExM_** | **Width _ExM_**  (μm) | **n _ExM_** | $\boldsymbol{F}_{\boldsymbol{1}\boldsymbol{D}}^{\boldsymbol{ExM}}$ (fold) |
| --- | --- | --- | --- | --- | --- |
| ***L. reuteri*** | 0.65 ± 0.06 | 24 |  | | |
|  | 0.60 ± 0.08 | 74 | 1.97 ± 0.41 | 44 | 3.3 |
|  |  |  | 1.75 ± 0.39 | 149 | 2.9 |
|  |  |  | 1.97 ± 0.24 | 36 | 3.3 |
|  |  |  | 1.44 ± 0.36 | 101 | 2.4 |
|  |  |  | 1.86 ± 0.39 | 106 | 3.1 |
|  |  |  | 1.52 ± 0.32 | 89 | 2.5 |
|  |  |  | 1.59 ± 0.32 | 101 | 2.6 |
|  |  |  |  |  | 3.0 ± 0.3 |
| ***E. faecalis*** | 0.62 ± 0.03 | 24 |  | | |
|  | 0.54 ± 0.09 | 60 | 1.73 ± 0.35 | 41 | 3.2 |
|  |  |  | 2.04 ± 0.30 | 51 | 3.7 |
|  |  |  | 2.60 ± 0.34 | 80 | 4.8 |
|  |  |  | 1.55 ± 0.24 | 51 | 2.8 |
|  |  |  | 1.42 ± 0.47 | 55 | 2.6 |
|  |  |  | 1.72 ± 0.17 | 74 | 3.2 |
|  |  |  | 1.98 ± 0.21 | 72 | 3.6 |
|  |  |  |  |  | 3.4 ± 0.7 |
| ***S. marcescens*** | 0.49 ± 0.04 | 24 |  | | |
|  | 0.57 ± 0.11 | 76 | 1.29 ± 0.20 | 30 | 2.3 |
|  |  |  | 1.25 ± 0.27 | 67 | 2.2 |
|  |  |  | 1.65 ± 0.32 | 29 | 2.9 |
|  |  |  | 1.72 ± 0.17 | 74 | 3.4 |
|  |  |  | 1.47 ± 0.38 | 65 | 2.6 |
|  |  |  | 1.46 ± 0.19 | 58 | 2.6 |
|  |  |  |  |  | 2.6 ± 0.4 |
| ***S. aureus*** | 0.82 ± 0.20 | 24 |  | | |
|  | 0.74 ± 0.15 | 62 | 1.50 ± 0.34 | 42 | 2.0 |
|  |  |  | 1.84 ± 0.26 | 13 | 2.5 |
|  |  |  | 1.79 ± 0.40 | 100 | 2.4 |
|  |  |  | 2.05 ± 0.50 | 79 | 2.8 |
|  |  |  | 1.80 ± 0.37 | 77 | 2.4 |
|  |  |  |  |  | 2.4 ± 0.3 |

* data of pre-ExM in grey cells were obtained from SEM micrographs as reference.

**Supplementary Table 3**. Codes for each dual-species biofilms. BS stands for Biofilm system.

| **Pair mixed biofilm** | | | **Biofilm system** |
| --- | --- | --- | --- |
| ***L. reuteri*** | **+** | ***E. faecalis*** | **BS1** |
| ***L. reuteri*** | **+** | ***S. marcescens*** | **BS2** |
| ***L. reuteri*** | **+** | ***S. aureus*** | **BS3** |
| ***E. faecalis*** | **+** | ***S. marcescens*** | **BS4** |
| ***E. faecalis*** | **+** | ***S. aureus*** | **BS5** |
| ***S. marcescens*** | **+** | ***S. aureus*** | **BS6** |

**Supplementary Table 4**. Widths and expansion factors of *Limosilactobacillus reuteri*, *Enterococcus faecalis*, *Serratia marcescens* and *Staphylococcus aureus* in dual-species biofilms. Values of Width_ExM_ represent mean average ± standard deviation.

| **Pair mixed specie** | **Width _ExM_(μm)** | | | **n_ExM_** | | | **Expansion (fold)** | | | $\bar{x}$ ± sd |
| --- | --- | --- | --- | --- | --- | --- | --- | --- | --- | --- |
| ***L. reuteri*** | 1.65 ± 0.39 | 1.94 ± 0.28 | 1.65 ± 0.44 | 94 | 69 | 58 | 2.7 | 3.2 | 2.7 | 2.9 ± 0.3 |
| ***E. faecalis*** | 2.00 ± 0.38 | 2.25 ± 0.26 | 2.15 ± 0.39 | 39 | 44 | 41 | 3.7 | 4.1 | 3.9 | 3.9 ± 0.2 |
| ***L. reuteri*** | 1.49 ± 0.19 | 1.91 ± 0.25 | 1.45 ± 0.37 | 50 | 52 | 37 | 2.6 | 3.3 | 2.5 | 2.8 ± 0.4 |
| ***S. marcescens*** |  |  |  |  |  |  |  |  |  |  |
| ***L. reuteri*** | 2.16 ± 0.43 | 2.08 ± 0.34 | 2.37 ± 0.48 | 34 | 36 | 39 | 3.6 | 3.4 | 3.9 | 3.6 ± 0.2 |
| ***S. aureus*** | 1.62 ± 0.31 | 1.55 ± 0.34 | 1.34 ± 0.60 | 52 | 51 | 61 | 2.2 | 2.1 | 1.8 | 2.0 ± 0.2 |
| ***E. faecalis*** | 1.58 ± 0.31 | 1.39 ± 0.32 | 1.27 ± 0.22 | 45 | 61 | 54 | 2.9 | 2.6 | 2.3 | 2.6 ± 0.3 |
| ***S. marcescens*** | 1.14 ± 0.18 | 1.20 ± 0.18 | 1.04 ± 0.23 | 18 | 9 | 63 | 2.0 | 2.1 | 1.8 | 2.0 ± 0.2 |
| ***E. faecalis*** | 1.95 ± 0.59 | 1.78 ± 0.33 | 1.62 ± 0.34 | 108 | 58 | 73 | 3.0 | 2.8 | 2.5 | 2.8 ± 0.4 |
| ***S. aureus*** |  |  |  |  |  |  |  |  |  |  |
| ***S. marcescens*** | 1.15 ± 0.25 | 1.70 ± 0.39 | 1.64 ± 0.23 | 27 | 37 | 45 | 2.0 | 3.0 | 2.9 | 2.6 ± 0.6 |
| ***S. aureus*** | 1.61 ± 0.30 | 1.94 ± 0.56 | 2.16 ± 0.42 | 47 | 46 | 38 | 2.2 | 2.6 | 2.9 | 2.6 ± 0.4 |

**Supplementary Table 5.** Widths (μm) of *Limosilactobacillus reuteri*, *Enterococcus faecalis*, *Serratia marcescens* and *Staphylococcus aureus* in mono- and dual-species biofilms measured by SEM or CLSM. Values represent mean average ± standard deviation.

|  |  | (2^nd^ specie) | | | |  |
| --- | --- | --- | --- | --- | --- | --- |
|  |  | ***L. reuteri*** | ***E. faecalis*** | ***S. marcescens*** | ***S. aureus*** | monospecies |
| 1^st^ specie | ***L. reuteri*** | 0.65 ± 0.06 | 0.62 ± 0.04 | 0.42 ± 0.06** | 0.51 ± 0.05 | 0.60 ± 0.80 |
|  | ***E. faecalis*** | 0.64 ± 0.04 | 0.62 ± 0.03 | 0.51 ± 0.05 | 0.57 ± 0.03 | 0.54 ± 0.09 |
|  | ***S. marcescens*** | 0.53 ± 0.05** | 0.48 ± 0.06 | 0.49 ± 0.04 | 0.57 ± 0.05 | 0.57 ± 0.11 |
|  | ***S. aureus*** | 0.77 ± 0.08 | 0.90 ± 0.10 | 0.77 ± 0.35* | 0.82 ± 0.20 | 0.74 ± 0.15 |
|  |  | **SEM** (*n=24*) | | | | **CLSM** |

* *n=7*.

*** Indiscernible species in co-cultured biofilms due to their similar morphology and size*
